# *Comprehensive in silico* analysis and molecular dynamics of the superoxide dismutase 1 (SOD1) variants related to amyotrophic lateral sclerosis

**DOI:** 10.1371/journal.pone.0247841

**Published:** 2021-02-25

**Authors:** Gabriel Rodrigues Coutinho Pereira, Bárbara de Azevedo Abrahim Vieira, Joelma Freire De Mesquita

**Affiliations:** 1 Department of Genetics and Molecular Biology, Bioinformatics and Computational Biology Laboratory, Federal University of the State of Rio de Janeiro (UNIRIO), Rio de Janeiro, Rio de Janeiro, Brazil; 2 Faculty of Pharmacy, Federal University of Rio de Janeiro (UFRJ), Rio de Janeiro, Rio de Janeiro, Brazil; Consejo Superior de Investigaciones Cientificas, SPAIN

## Abstract

Amyotrophic Lateral Sclerosis (ALS) is the most frequent motor neuron disorder, with a significant social and economic burden. ALS remains incurable, and the only drugs approved for its treatments confers a survival benefit of a few months for the patients. Missense mutations in superoxide dismutase 1 (SOD1), a major cytoplasmic antioxidant enzyme, has been associated with ALS development, accounting for 23% of its familial cases and 7% of all sporadic cases. This work aims to characterize *in silico* the structural and functional effects of SOD1 protein variants. Missense mutations in SOD1 were compiled from the literature and databases. Twelve algorithms were used to predict the functional and stability effects of these mutations. ConSurf was used to estimate the evolutionary conservation of SOD1 amino-acids. GROMACS was used to perform molecular dynamics (MD) simulations of SOD1 wild-type and variants A4V, D90A, H46R, and I113T, which account for approximately half of all ALS-SOD1 cases in the United States, Europe, Japan, and United Kingdom, respectively. 233 missense mutations in SOD1 protein were compiled from the databases and literature consulted. The predictive analyses pointed to an elevated rate of deleterious and destabilizing predictions for the analyzed variants, indicating their harmful effects. The ConSurf analysis suggested that mutations in SOD1 mainly affect conserved and possibly functionally essential amino acids. The MD analyses pointed to flexibility and essential dynamics alterations at the electrostatic and metal-binding loops of variants A4V, D90A, H46R, and I113T that could lead to aberrant interactions triggering toxic protein aggregation. These alterations may have harmful implications for SOD1 and explain their association with ALS. Understanding the effects of SOD1 mutations on protein structure and function facilitates the design of further experiments and provides relevant information on the molecular mechanism of pathology, which may contribute to improvements in existing treatments for ALS.

## Introduction

Amyotrophic Lateral Sclerosis (ALS) is a highly disabling, progressive, and fatal neurodegenerative disorder characterized by injury and death of upper motor neurons in the cerebral cortex and lower motor neurons in the brain stem and spinal cord [1]. ALS is characterized by the progressive loss of voluntary motor activity, which impairs the patient’s ability to work and perform daily activities [2], usually resulting in the patient’s death due to respiratory paralysis within three to four years after the symptoms’ onset [3]. The patient’s illness-related absence from work, caregiver needs, and the medical costs of ALS represent a significant economic burden, which has been estimated at over one billion dollars a year only in the United States [4]. ALS is the most frequent motor neurodegenerative disorder in adults [5], affecting more than 220,000 people worldwide. Due to population aging, the number of people affected by ALS is projected to increase by 69% in the next 20 years, particularly among developing countries [6].

Despite its importance, ALS remains uncurable [7]. The only drugs approved for the treatment of ALS, Riluzole, and Edaravone, confers a survival benefit of a few months for the patients [8]. Thus, there is an urgent need to develop more effective treatments for diseases such as ALS, but these will only come with a deep understanding of their causes and associated mechanisms [9]. Due to its heterogeneous and complex nature, the exact pathway that causes ALS degeneration is not yet fully understood [10]. However, glutamate cytotoxicity, inflammatory pathway, oxidative stress, and protein aggregation are among the main mechanisms associated with ALS development [11].

ALS is classified as sporadic (sALS) and familial (fALS). Most ALS cases are sporadic (95–90%), while 5–10% are familial [12]. Mutations in the *SOD1 gene* are frequent and important causes of ALS, accounting for 23% of the fALS cases, and approximately 7% of all sALS cases [13]. The SOD1 gene codes superoxide dismutase 1 (SOD1), a major cytoplasmic antioxidant enzyme. SOD1 is a Cu/Zn metalloenzyme that catalyzes the dismutation of superoxide radicals (O_2_-.) to hydrogen peroxide (H_2_O_2_) and molecular oxygen (O_2_) [14]. This mechanism protects cells from the harmful effects of superoxide radicals and, consequently, from oxidative stress [15]. More than 185 SOD1 mutations have already been associated with ALS [13]. Although these mutations affect different locations within the SOD1 structure [16], many of them lead to protein-aggregation and increased oxidative damage, which are central events in ALS pathogenicity [17, 18].

The computational approach has become widely used to characterize the effects of mutations in proteins and to identify potentially deleterious mutations, particularly because of its relatively high cost/efficiency and accuracy [19]. We applied computational predictions to the study of SOD1 protein variants following the methodology previously established by our group [16, 19–22]. This work aims to characterize the effects of these variants on the SOD1 structure, which could assist the design of future experiments and provide relevant information on the molecular mechanism of pathology that may lead to improvements in existing treatments for ALS [22–24].

## Materials and methods

### Dataset

The structure, sequence, and natural variants of SOD1 protein were retrieved from the Protein Data Bank (PDB) [25], UniProt [26], OMIM [27], ALSoD [28], dbSNP [29], ClinVar [30], and PubMed [27].

### Functional and stability prediction analysis

The functional and stability effects of the SOD1 protein variants were predicted using the following algorithms: SNAP2, SNPs&GO, PolyPhen2, PMUT, PhD-SNP, MutPred2 [19], Panther [31], Provean [32], MAPP [33], I-Mutant3.0 [34], SIFT and SNPEffect4.0 [35, 36].

### Evolutionary conservation analysis

The evolutionary conservation analysis of human SOD1 was performed using the ConSurf server, which estimated the evolutionary conservation degree of each SOD1 amino acid [37]. The following parameters were selected for this analysis: PDB ID: 2C9V; Chain identifier: A; homologous search algorithm: PSI-BLAST; number of iterations: 3; E-value cut-off: 0.0001; protein database: UniProt; reference sequence: closest; number of reference sequences selected: 150; maximum sequence identity: 95%; minimum identity for counterparts: 35%; alignment method: MAFFT-L-INS-i; calculation method: Bayesian; evolutionary substitution model: best model (standard).

### Molecular dynamics simulations

Molecular Dynamics (MD) simulations of the wild-type SOD1 protein and its variants A4V, D90A, H46R, and I113T were performed using the GROMACS 2018.8 package [38]. The software Visual Molecular Dynamics 1.9.2 was used to induce the mutations A4V, D90A, H46R, and I113T on the crystallographic structure of wild-type SOD1 (PDB ID: 2C9V) [25]. The variants A4V, D90A, H46R, and I113T were selected for the MD simulations because they account for approximately half of all ALS-SOD1 cases in the United States [39], Europe, Japan, and United Kingdom [15], respectively.

The MD simulations were performed in triplicates according to the methodology described by Pereira, Tellini, and De Mesquita, 2019 [22]. The force-field AMBER99SB-ILDN was selected for the simulations. The molecules were solvated in a dodecahedral box with TIP3P water molecules, neutralized by adding Na^+^Cl^-^ ions, and minimized using the steepest descent method. After system minimization, an NVT ensemble (constant number of particles, volume, and temperature) followed by an NPT ensemble (constant number of particles, pressure, and temperature) were performed for 100ps at a pressure of 1atm and temperature of 300K. The V-rescale thermostat and Parrinello-Rahman barostat were selected for the NVT and NPT ensembles. The production simulations were further performed at a temperature of 300K for a duration of 300ns using the LINCS (linear constraint solver) and PME (particle mesh Ewald) algorithms.

The MD trajectories were analyzed using the following GROMACS distribution programs: *gmx rms*, *gmx rmsf*, *gmx gyrate*, *gmx dssp*, *gmx sasa*, and *gmx hbond*. These MD analyses generated parameters values for root-mean-square deviation (RMSD), root-mean-square fluctuation (RMSF), B-factor, radius of gyration (Rg), solvent accessible surface area (SASA), and secondary structure (SS). The MD trajectories were also submitted to the Bio3D library in R software [40], which was used to perform principal component analysis (PCA) of the wild-type SOD1 and its variants. PCA was performed for the Cα atoms of protein structure, and their Cartesian coordinates were used to generate the covariance matrices.

## Results

### Dataset

**Fig 1:**
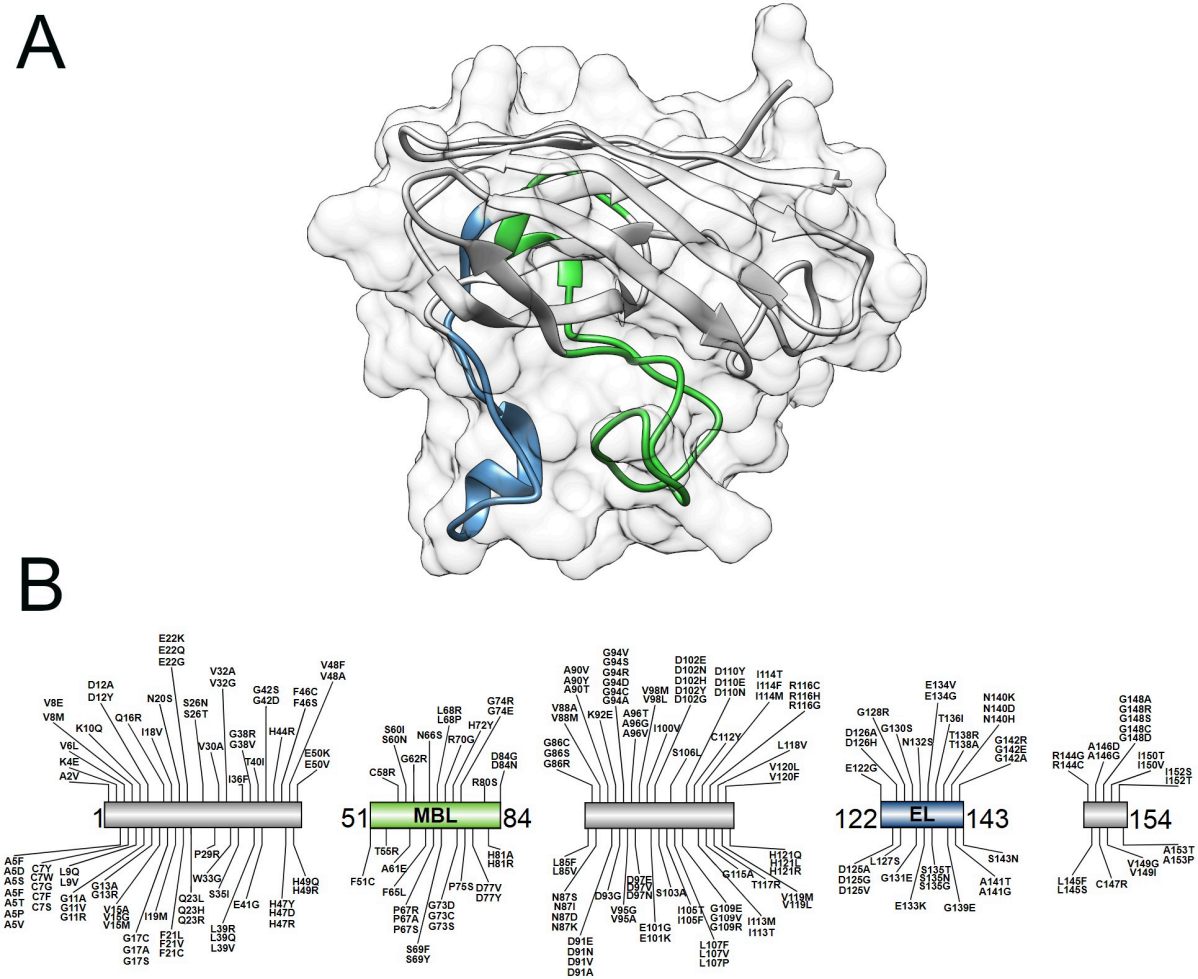
Crystallographic structure and schematic representation of SOD1 protein and its variants. The metal-binding (amino-acids 51–84) and electrostatic loops (amino-acids 122–143) of SOD1 are represented in green and blue, respectively. (A) Secondary structure representation of the crystallographic structure of SOD1 (PDB ID: 2C9V). The protein’s surface is also shown. (B) Schematic representation of the SOD1 protein sequence and its 233 variants compiled from databases and literature.

The complete crystallographic structure of wild-type SOD1 retrieved from the Protein Data Bank [PDB ID: 2C9V] is shown in Fig 1A. The 2C9V structure was selected because it has the highest atomic resolution (1.07Å) among the SOD1 structures available in PDB [25]. The complete amino-acid sequence of the SOD1 protein was retrieved from the UniProt database [UniProt ID: P00441] [26]. SOD1 contains an 8-stranded beta-barrel motif and several loops. Two loop elements termed metal-binding (amino-acids 51–84) and electrostatic loops (amino-acids 122–143) project from the beta-barrel (Fig 1A) and are important for the SOD1 enzymatic activity [39]. The electrostatic loop is composed of positively charged amino-acids that guide superoxide radicals into the SOD1 active site where the copper ion is located [41]. The metal-binding loop coordinates the binding of Zinc and Copper ions [42, 43], which is structurally important for SOD1 [44]. These loops together shape the active site pocket [43].

Two hundred and thirty-three (233) mutations in SOD1 protein were compiled from the databases and literature consulted (S1 Table). As shown in Fig 1B, SOD1 mutations occur all over the protein structure. Thirty-one (31) mutations were found occurring at the metal-binding loop, while 30 mutations were found at the electrostatic loop of SOD1.

### Functional and stability prediction analysis

**Fig 2:**
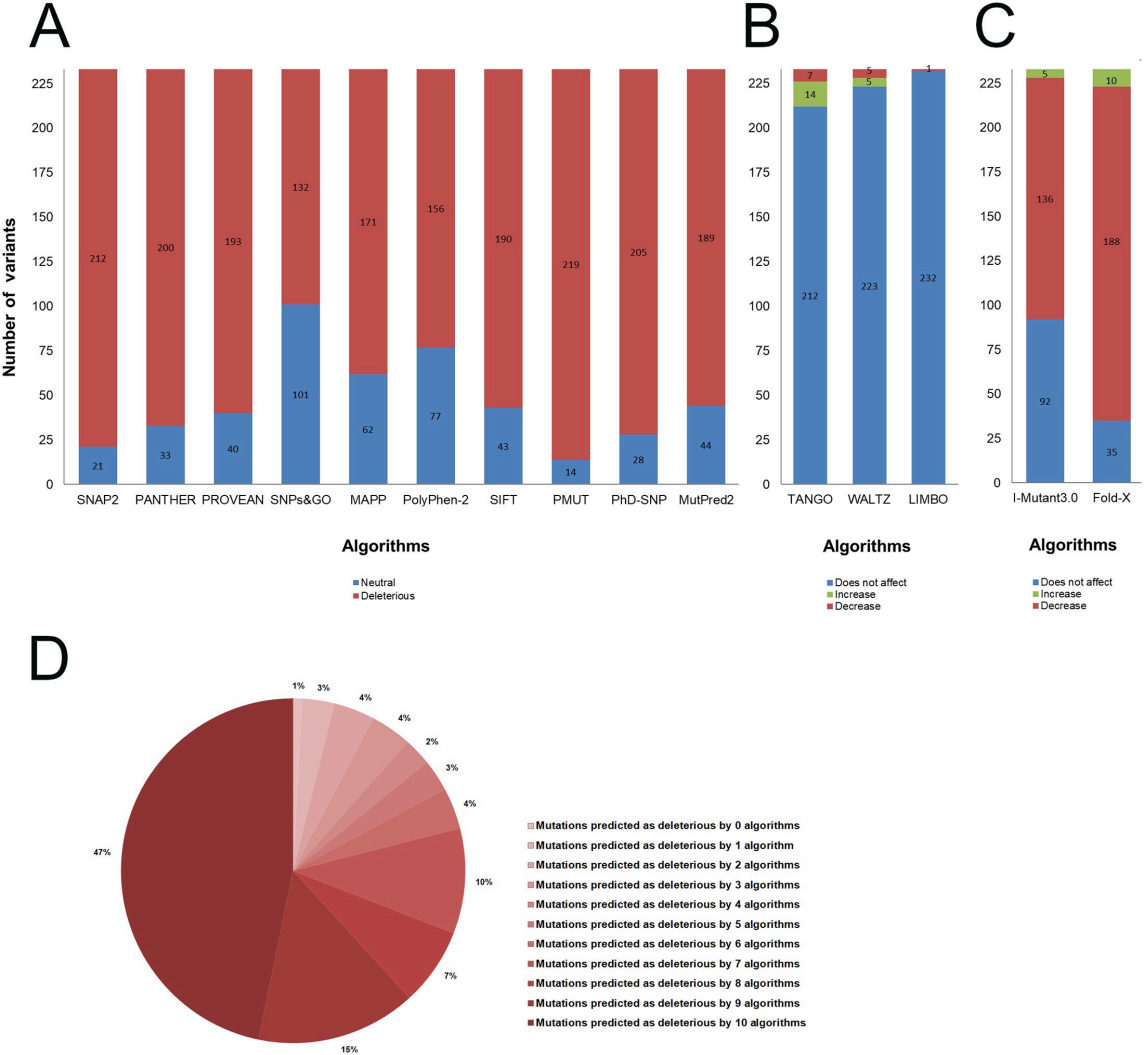
Functional and stability predictions of SOD1 protein variants. The SOD1 variants compiled from the literature and databases (233) were analyzed using functional and stability prediction algorithms. (A) Functional prediction using ten different algorithms. The bar plot indicates the number of neutral (blue) and deleterious (red) predictions for each algorithm. (B) Prediction of aggregation propensity (TANGO), amyloid propensity (WALTZ), and chaperone binding (LIMBO) using the SNPEffect4.0 algorithm. The bar plot indicates the number of variants that increase (green), decrease (red), and do not affect (blue) the parameter analyzed. (C) Stability prediction using the FoldX and I-Mutant3.0 algorithms. The bar plot indicates the number of variants that increase (green), decrease (red), and do not affect (blue) protein stability. (D) The circle chart indicates the percentage of variants predicted as deleterious by zero to ten algorithms.

As shown in Fig 2A, all the algorithms used predicted more than 50% of the SOD1 mutations as deleterious. SNPs&GO was the algorithm with the lowest number of deleterious predictions, while PMUT was the algorithm with the highest number of deleterious predictions (Fig 2A).

As shown in Fig 2D, 47% of the analyzed variants were predicted as deleterious by all the ten functional prediction algorithms used, while only 1% of them were predicted as neutral by all the algorithms. Moreover, the SOD1 protein variants presented an elevated rate of deleterious predictions (Fig 2D). This analysis also showed that the mutations with the highest rates of deleterious predictions were concentrated in the metal-binding (amino-acids 51–84) and electrostatic (amino-acids 122–143) loops of SOD1 (S2 Table).

According to the SNPEffect4.0, twenty-one variants were predicted to affect the SOD1 aggregation tendency, ten variants were predicted to affect amyloid propensity, and one variant was predicted to affect chaperone binding (Fig 2B). The variants predicted to affect protein aggregation mostly occur in the N and C-terminal portions of SOD1, while the variants predicted to affect amyloid propensity mostly occur in its N-terminal portion (S3 Table).

As shown in Fig 2C, each algorithm used predicted more than 50% of the SOD1 variants as decreasing protein stability. This analysis also showed that 49% of these variants were predicted to decrease protein stability by I-Mutant and Fold-X, while less than 6% were predicted by both algorithms to increase or not affect this feature (S3 Table). The I-Mutant and Fold-X predictions were concordant for 54.5% of the analyzed variants, but only 2.2% of all predictions were opposed (i.e. increase and decrease for the same variant).

The variants A4V, D90A, H46R, and I113T, which are the most frequent ALS-related mutations in SOD1 [15, 39], were predicted as deleterious by 90%, 50%, 100%, and 100% of the algorithms used, respectively (S1 Fig). The variants A4V, D90A, and I113T were also predicted to decrease protein stability by Fold-X and I-Mutant, while the variant H46R was only predicted as destabilizing by Fold-X. Furthermore, the A4V variant was predicted to increase protein aggregation. None of these variants were predicted to affect amyloid propensity and chaperone binding (S3 Table).

### Evolutionary conservation analysis

**Fig 3:**
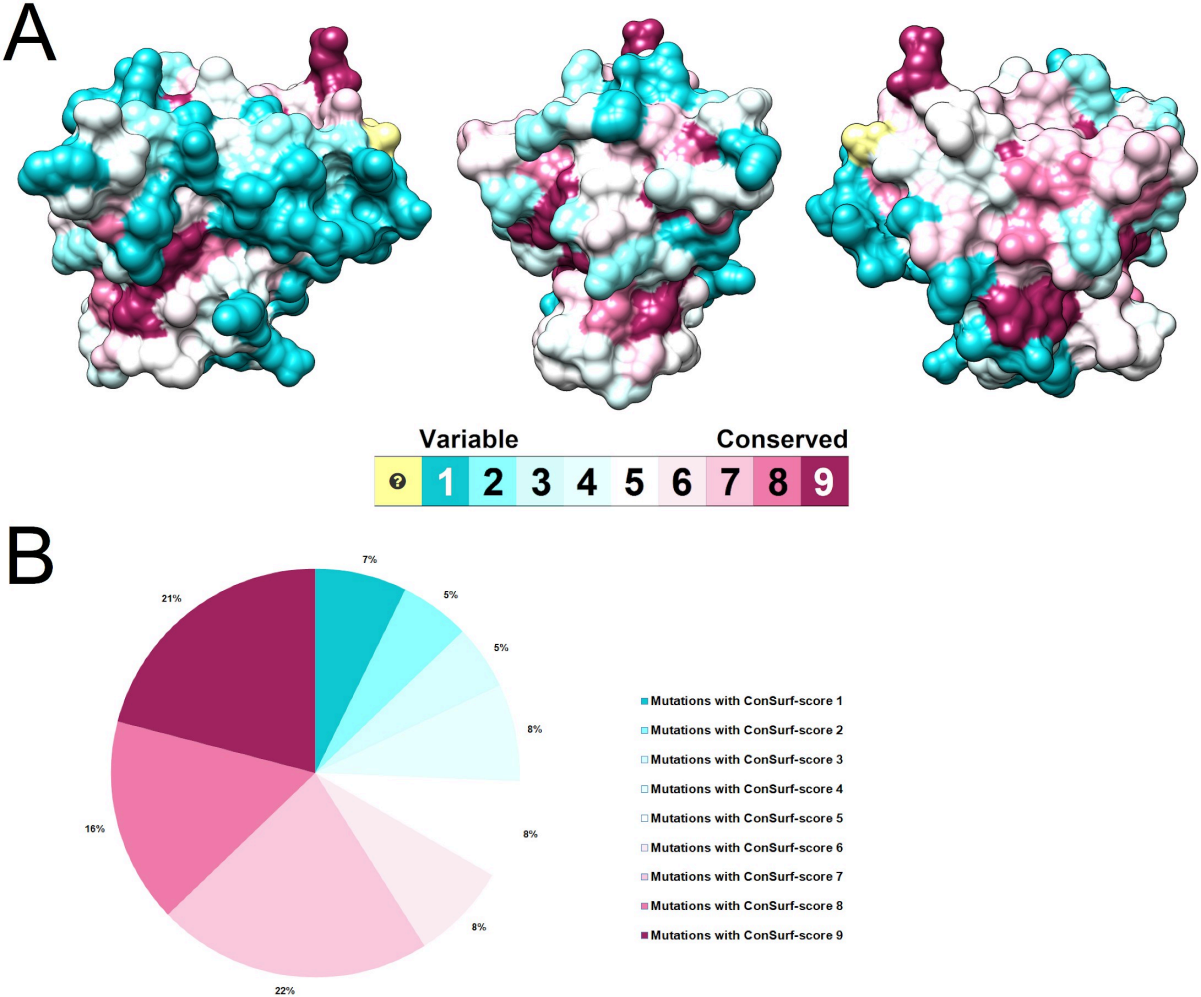
Evolutionary conservation analysis of SOD1 protein. (A) The evolutionary conservation profile of SOD1 is shown in three different angles, displaying different sides of the protein. Each SOD1 amino-acid is represented as a surface model and colored according to its ConSurf conservation score. The color-coding bar shows the ConSurf coloring scheme, which varies from (1) cyan and variable to (9) maroon and conserved. The amino-acids colored yellow did not receive conservation scores due to insufficient data. (B) The circle chart indicates the percentage of variants affecting amino-acids predicted as variable to conserved. The circle chart is also colored according to the ConSurf coloring scheme.

The crystallographic structure of wild-type SOD1 (PDB ID: 2C9V) was submitted to the ConSurf server, which estimated the evolutionary conservation score (ConSurf-score) of each protein’s amino acid. ConSurf performs multiple-sequence alignments to compare a given amino acid sequence to its homologous sequences and estimate their evolutionary conservation. ConSurf is useful for identifying functionally important regions on proteins, as these are usually conserved throughout evolution [37]. The computed ConSurf-score was projected on the protein’s surface (Fig 3A). The SOD1 amino-acids were colored according to their ConSurf-score following a coloring-code scheme, which varies from (1) cyan and variable to (9) maroon and conserved.

As shown in Fig 3B, 59% of the analyzed SOD1 variants occur in conserved positions (i.e. ConSurf-score ≥ 7), while only 17% of them occur in variable positions (i.e. ConSurf-score ≤ 3). This analysis also showed that variants affecting highly conserved positions (i.e. ConSurf-score = 9) mainly occur at the metal-binding (amino-acids 51–84) and electrostatic (amino-acids 122–143) loops of SOD1. Among the most frequent ALS-related mutations in SOD1 [15, 39], the variant I113T was the only variant predicted to affect a variable region. The variants A4V and H46R were predicted to affect conserved amino-acids of SOD1, while the variant D90A was predicted to affect an average conserved position (S2 Fig).

### Molecular dynamics simulations

MD is a computational method that predicts the time-dependent motion of an atomic system by solving Newton’s equations of motion [45]. The MD simulations can be used to reproduce the real behavior of a protein in its environment, which allows the study of relevant biomolecular processes, including conformational change, protein folding, and ligand binding. These simulations can also be used to predict the effects of perturbations such as mutation, phosphorylation, and protonation on the protein’s structure [46]. During an MD, the interatomic interactions are calculated at every simulation step and the resulting atomic positions are registered in a trajectory file, which provided detailed information on changes in protein conformation that can be used to assess several structural parameters [19].

We performed MD simulations of the wild-type SOD1 (PDB ID: 2C9V) and its most frequent ALS-related variants, i.e. A4V, D90A, H46R, and I113T [15, 39], to better understand the impact of these amino-acid substitutions on protein structure. The following structural parameters were analyzed from the MD trajectories: RMSD, RMSF, B-factor, Rg, SASA, SS, and PCA.

**Fig 4:**
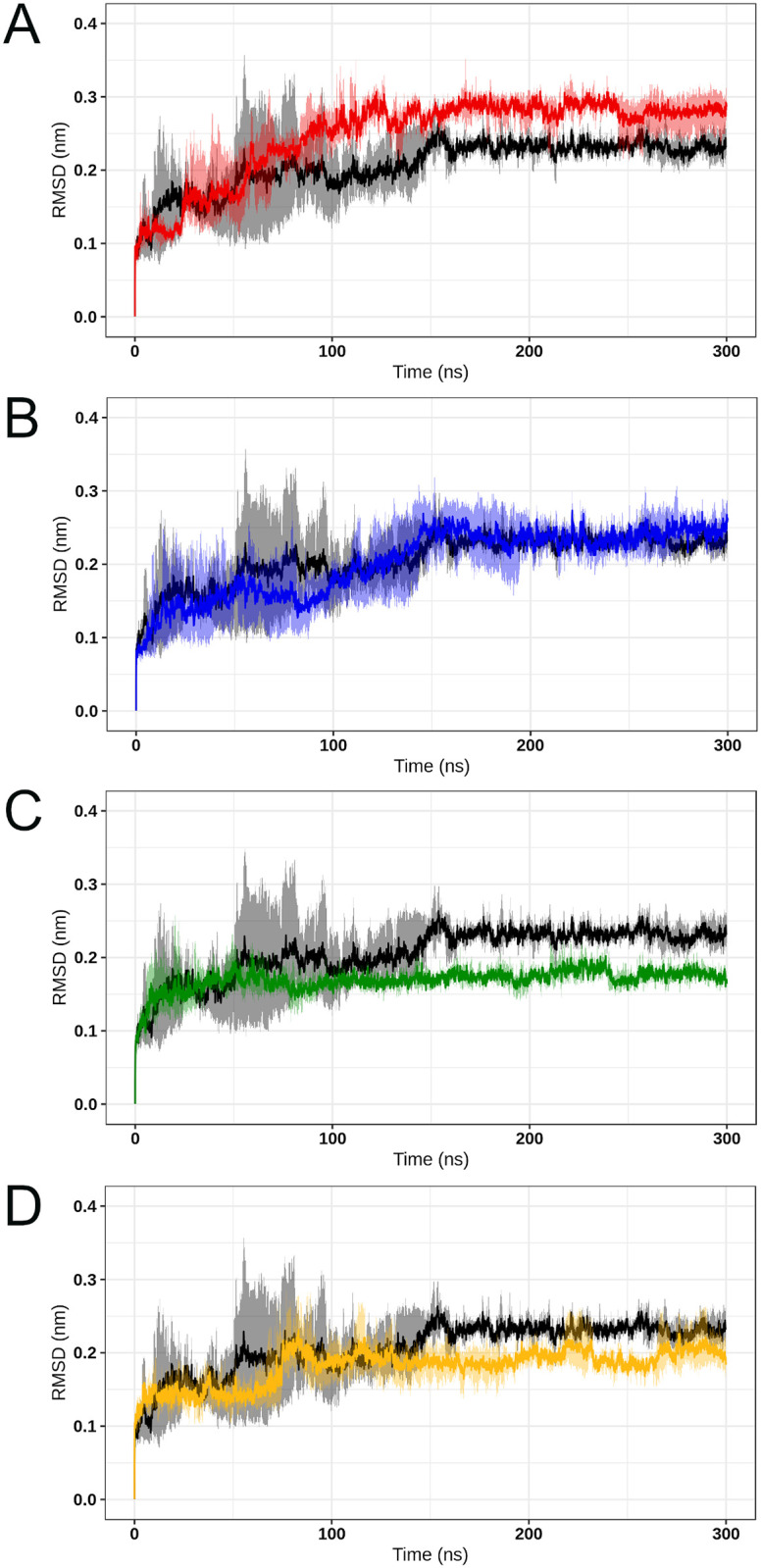
RMSD analysis of wild-type SOD1 and its variants. The RMSD values computed for the backbone atoms of wild-type SOD1 and its variants at 300K are shown over time. The means (solid lines) and confidence intervals (smooth lines) are displayed for the triplicates. (A) The wild-type is represented in black, while variant A4V is represented in red. (B) The wild-type is represented in black, while variant D90A is represented in blue. (C) The wild-type is represented in black, while variant H46R is represented in green. (D) The wild-type is represented in black, while variant I113T is represented in yellow.

RMSD is commonly used in MD simulations to measure the spatial differences between a starting structure and its subsequent coordinates computed over time [47]. This parameter is useful to analyze the time-dependent motion of protein structures and to determine their structural convergence throughout the simulation [19]. RMSD values were calculated from the total number of protein conformations computed in the MD trajectories. As shown in Fig 4, triplicates for wild-type SOD1 and its variants presented a similar behavior throughout the simulation, given their respective means and confidence intervals. A sudden increase in RMSD values was observed at the beginning of all simulations. This was followed by the establishment of a plateau in RMSD values after approximately 150ns, which suggests that the protein structures float around average stable conformations [16]. The initial effects of the trajectories, i.e. those occurring until 150ns, were discounted in further analyses for meaningful comparison [19, 34].

**Fig 5:**
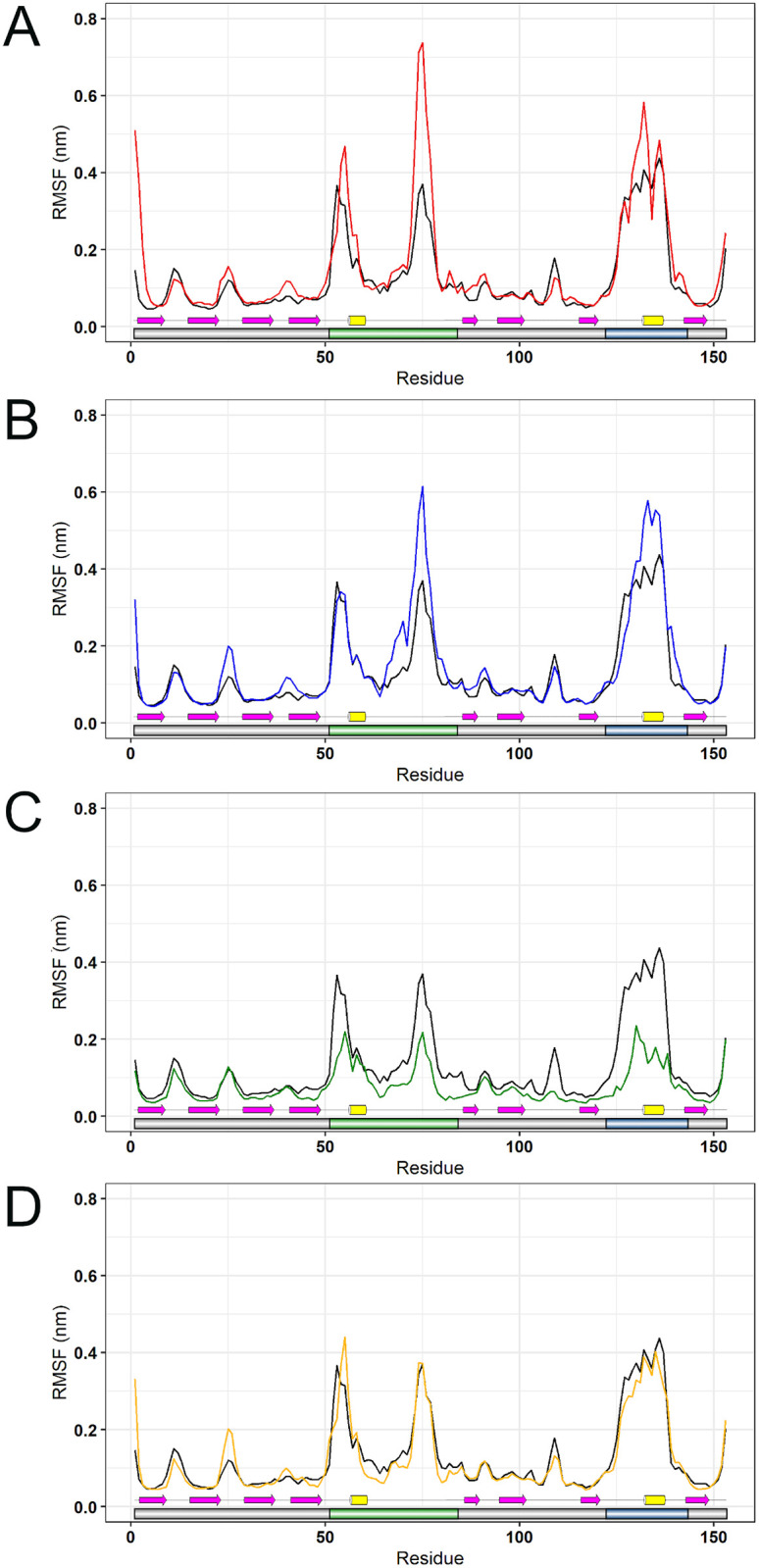
RMSF analysis of wild-type SOD1 and its variants. The RMSF values for each residue of wild-type SOD1 and its variants at 300K are shown as a line plot. Schematic representations of the SOD1 functional regions and secondary structure are shown to further comparison. The metal-binding and electrostatic loops of SOD1 are highlighted in green and blue, respectively. β-strands are represented by yellow barrels, α-helices are represented by magenta arrows, and the coils are represented by the thin black lines. (A) The wild-type is represented in black, while variant A4V is represented in red. (B) The wild-type is represented in black, while variant D90A is represented in blue. (C) The wild-type is represented in black, while variant H46R is represented in green. (D) The wild-type is represented in black, while variant I113T is represented in yellow.

RMSF is a measure of the structural displacement of an amino-acid from its average position throughout the simulation [16]. RMSF is useful to assess local flexibility in protein structures, allowing the identification of flexible and rigid regions [48]. The RMSF analysis pointed to a flexibility increase at the metal-binding and electrostatic loops of the variants A4V and D90A, in addition to a flexibility decrease in the same regions of H46R (Fig 5).

**Fig 6:**
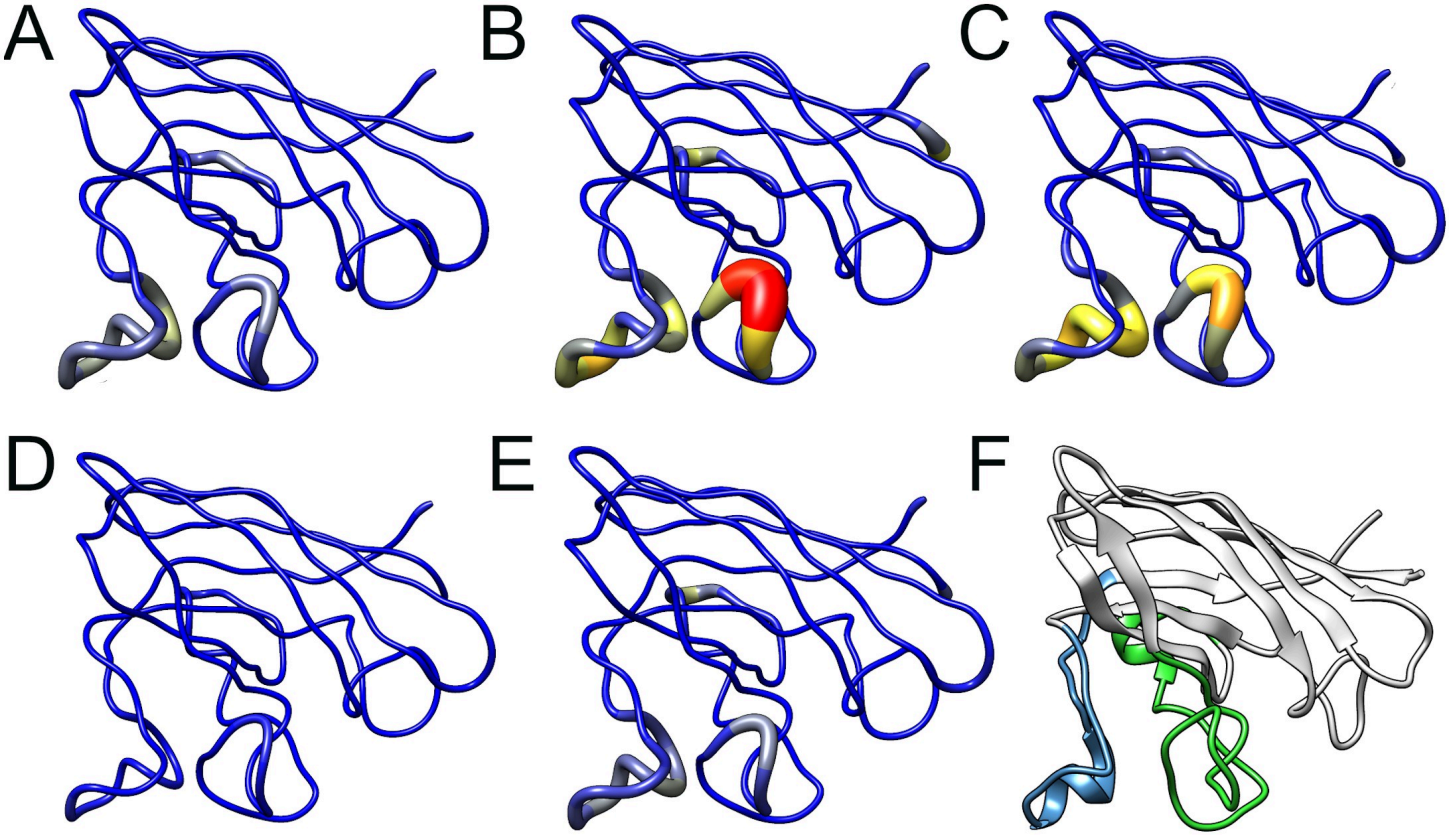
B-Factor analysis of wild-type SOD1 and its variants. The amino-acids residues of wild-type SOD1 (A) and its variants A4V (B), D90A (C), H46R (D), and I113T (E) are sized and colored according to their B-factor values, following a coloring-thickness scale that varies from blue and thin (rigid residues) to red and thick (flexible residues). The three-dimensional representation of SOD1 and its functional regions (F) are also shown to further comparison. The metal-binding and electrostatic loops are colored in green and blue, respectively.

Flexibility can also be assessed during an MD simulation by analyzing the B-factor, a temperature-displacement factor. B-factor is a measure of the structural displacement of an amino-acid around its average position due to thermal vibrations [49]. The B-factor values computed for each SOD1 amino-acid were projected on the protein surface, providing an interesting three-dimensional representation of structural flexibility [19]. The B-factor distribution over a protein structure also provides an important indicator of its dynamics [50]. As shown in Fig 6, the variants A4V and D90A presented increased flexibility at their metal-binding and electrostatic loops, while the variant H46R presented decreased flexibility at those regions when compared to the wild-type SOD1. The B-factor analysis suggested flexibility alterations in the variants A4V, D90A, and H46R at regions similar to those found altered in the RMSF analysis.

PCA is also known as essential dynamics (ED) when applied to analyze MD simulations [19]. ED linear transforms the high-dimensional and complex data contained in a molecular trajectory into a low-dimensional space in which the large-scale protein motions occur [51], reducing the number of dimensions needed to describe protein dynamics. This statistical technique systematically filters the observed motions from the largest to smallest spatial-scale in a molecular trajectory using a covariance matrix constructed with the Cartesian coordinates representing atomic positions [52]. ED allows separating the essential from the remaining protein motions. The essential motions (i.e. the largest-scales motions) are usually biologically relevant movements such as opening, closing, and flexing, while the remaining motions describe small irrelevant local fluctuations [20]. The essential motions are often confined into the first two PCA modes (principal components) [53].

**Fig 7:**
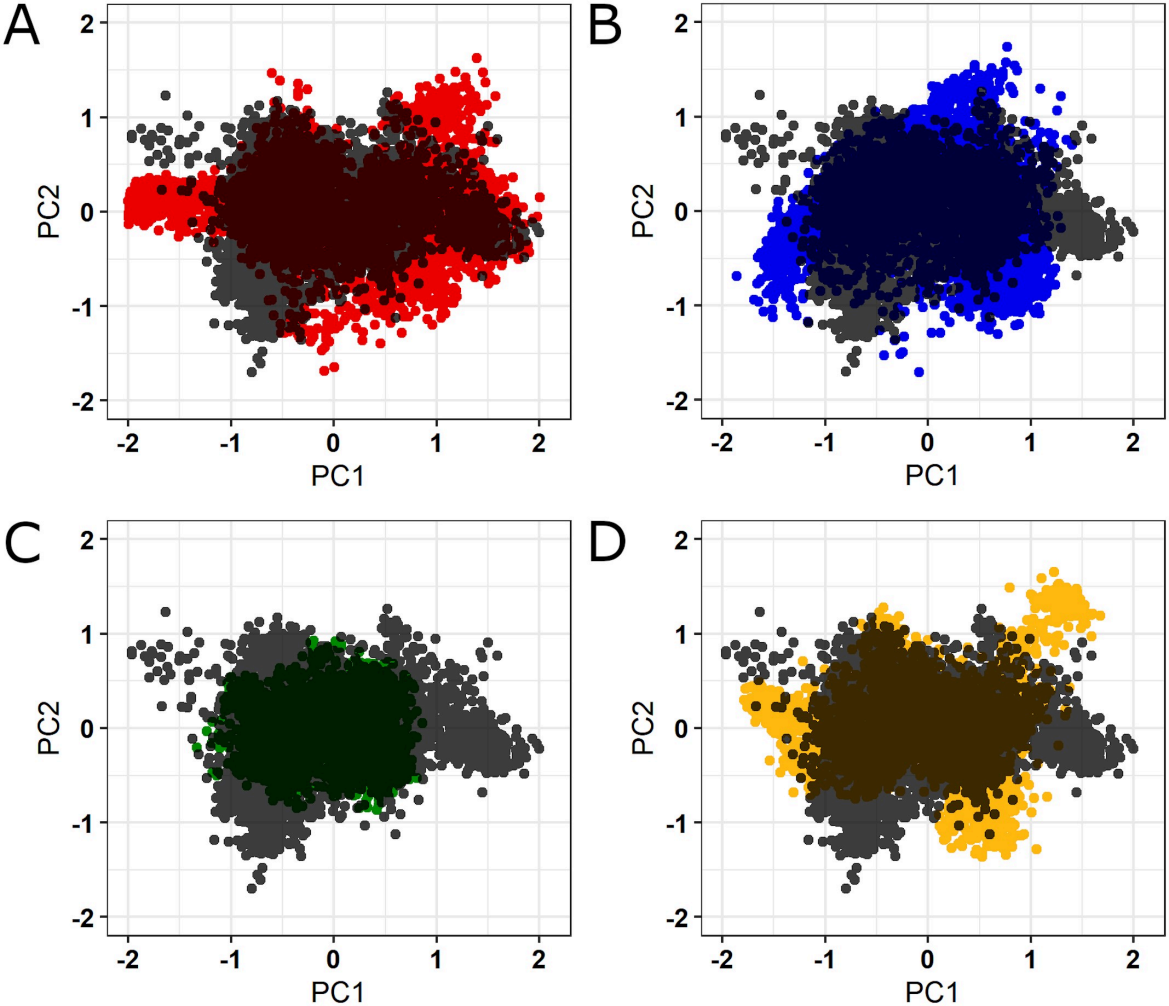
PCA for the wild-type SOD1 and its variants. Projections for the first two principal components extracted from the MD trajectories. (A) Comparison between the PCA projections for the wild-type SOD1 (black) and its variant A4V (red). (B) Comparison between the PCA projections for the wild-type SOD1 (black) and its variant D90A (blue). (C) Comparison between the PCA projections for the wild-type SOD1 (black) and its variant H46R (green). (D) Comparison between the PCA projections for the wild-type SOD1 (black) and its variant I113T (yellow).

The projections for the MD trajectories of wild-type SOD1 and its variants into the subspace spanned by PC1 and PC2 are shown in Fig 7. The ED analysis suggested that the first two principal components (PC1 and PC2) capture the dominant motions, accounting for 62.8%, 73.3%, 70.7%, 35.3%, and 62.1% of the total variance for wild-type SOD1 and its variants A4V, D90A, H46R, and I113T, respectively. As shown in Fig 7, the variants A4V, D90A, and I113T occupied a higher area in conformational space, while the variant H46R occupied a smaller area. Changes in cluster shape were also observed in the conformational space of all variants when compared to the wild-type. The ED analysis thus pointed to alterations in the overall essential dynamics of all variants.

**Fig 8:**
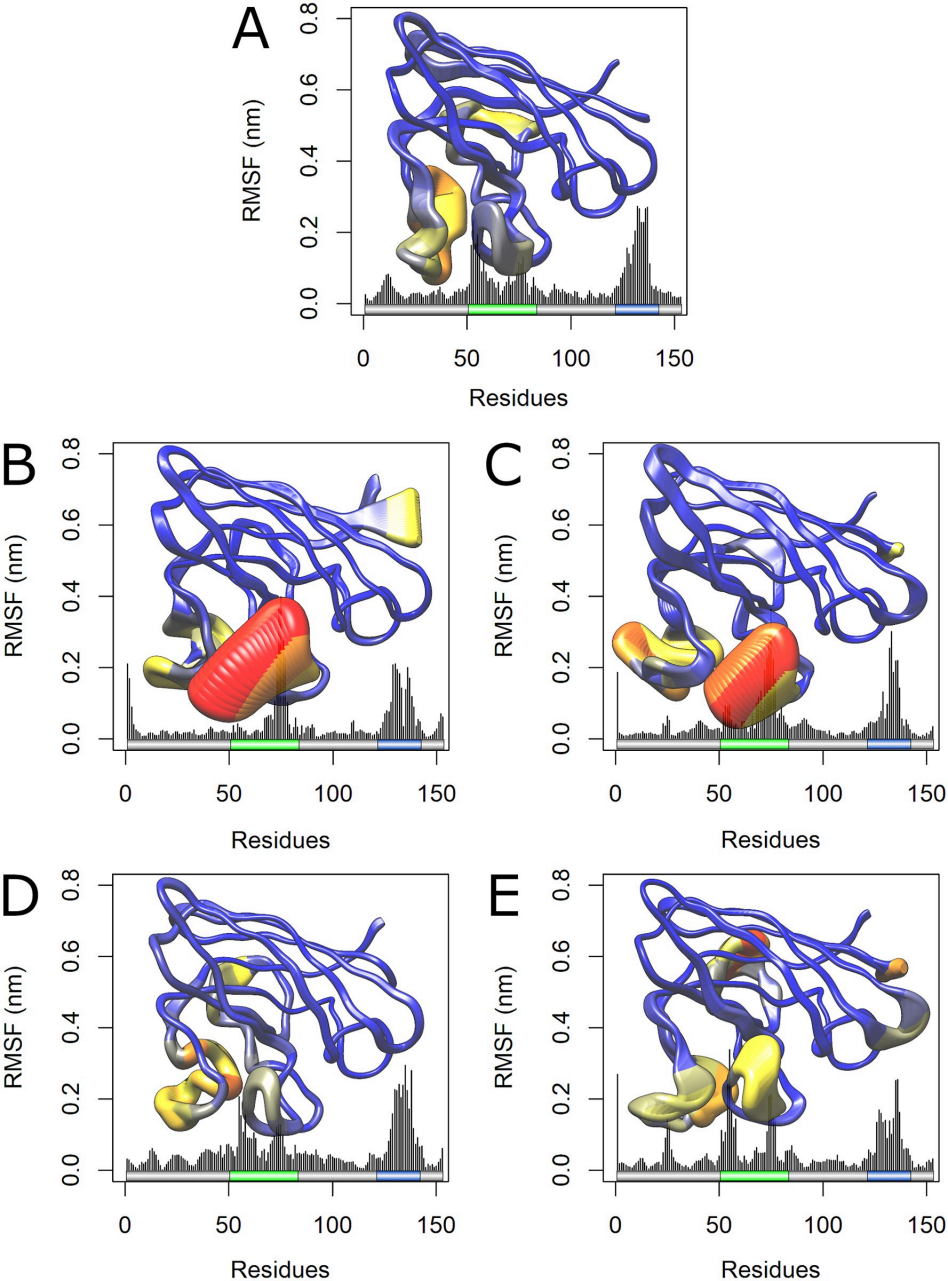
RMSF contribution to PC1 of wild-type SOD1 and its variants. The RMSF contribution of each amino-acid to PC1 is shown as a line plot and projected on the corresponding structure. Schematic representations of the SOD1 functional regions and are shown to further comparison. The metal-binding and electrostatic loops of SOD1 are highlighted in green and blue, respectively. Each amino-acid of SOD1 wild-type and variants was colored and sized according to its RMSF contribution, following a coloring-thickness scale that varies from blue and thin (low fluctuations) to red and thick (high fluctuations). (A) RMSF contribution of wild-type SOD1 to PC1. (B) RMSF contribution of variant A4V to PC1. (C) RMSF contribution of variant D90A to PC1. (D) RMSF contribution of variant H46R to PC1. (E) RMSF contribution of variant I113T to PC1.

**Fig 9:**
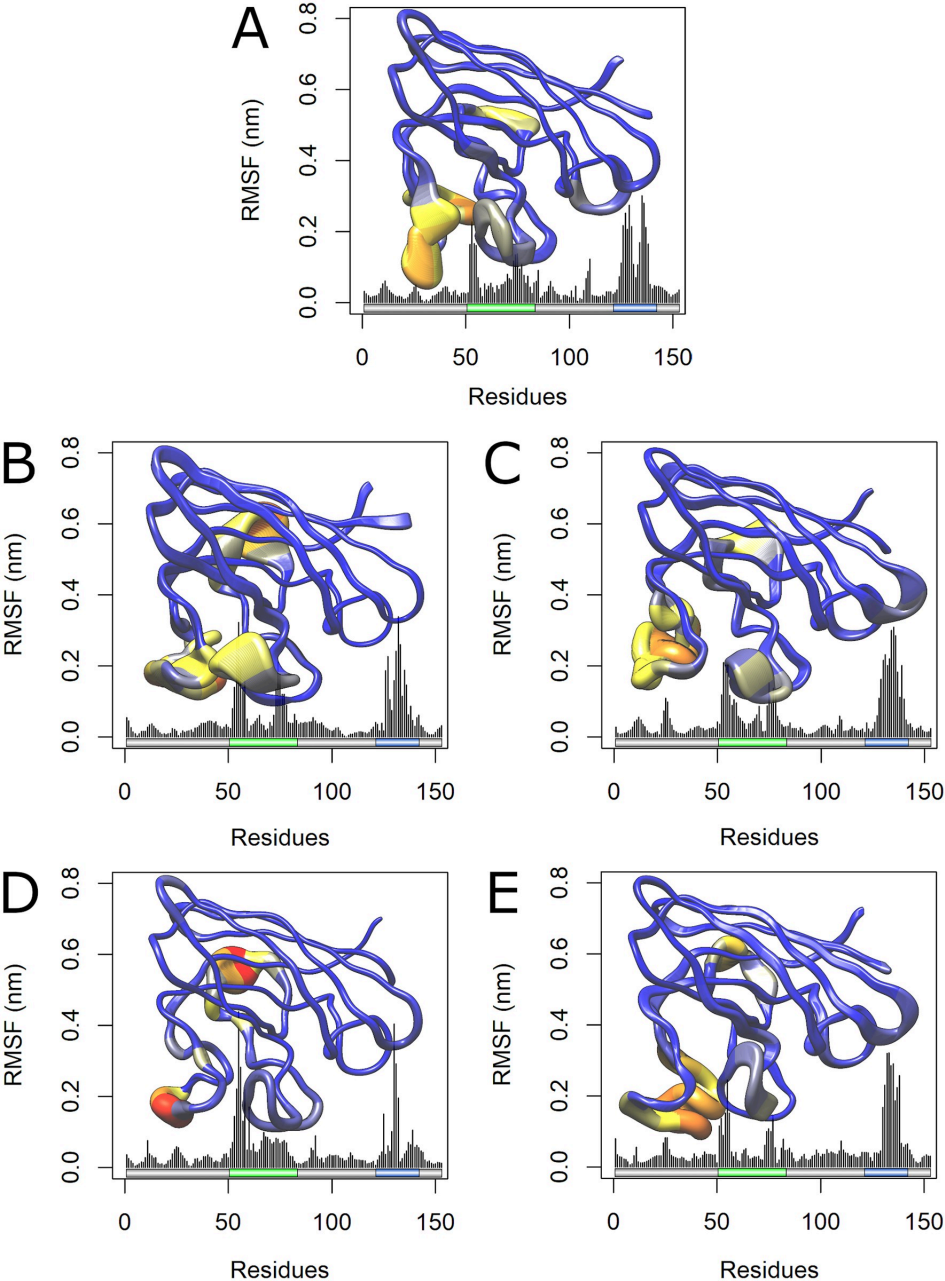
RMSF contribution to PC2 of wild-type SOD1 and its variants. The RMSF contribution of each amino-acid to PC2 is shown as a line plot and projected on the corresponding protein structure. Schematic representations of the SOD1 functional regions are also shown to further comparison. The metal-binding and electrostatic loops of SOD1 are highlighted in green and blue, respectively. Each amino-acid of SOD1 wild-type and variants was colored and sized according to its RMSF contribution, following a coloring-thickness scale that varies from blue and thin (low fluctuations) to red and thick (high fluctuations). (A) RMSF contribution of wild-type SOD1 to PC2. (B) RMSF contribution of variant A4V to PC2. (C) RMSF contribution of variant D90A to PC2. (D) RMSF contribution of variant H46R to PC2. (E) RMSF contribution of variant I113T to PC2.

We also analyzed the RMSF contribution of each protein amino-acid for the first two principal components. RMSF values for the PC1 and PC2 projections are shown in Figs 8 and 9, respectively. The RMSF contribution to PC1 pointed to increased essential mobility at the metal-binding loops of the A4V, D90A, and I113T (Fig 8), while the RMSF contribution for the PC2 pointed to essential mobility alterations at the metal-binding and electrostatic loop of the A4V and H46R variants (Fig 9). As shown in Fig 8, these alterations are particularly high at the metal-binding loop of A4V and D90A variants.

**Fig 10:**
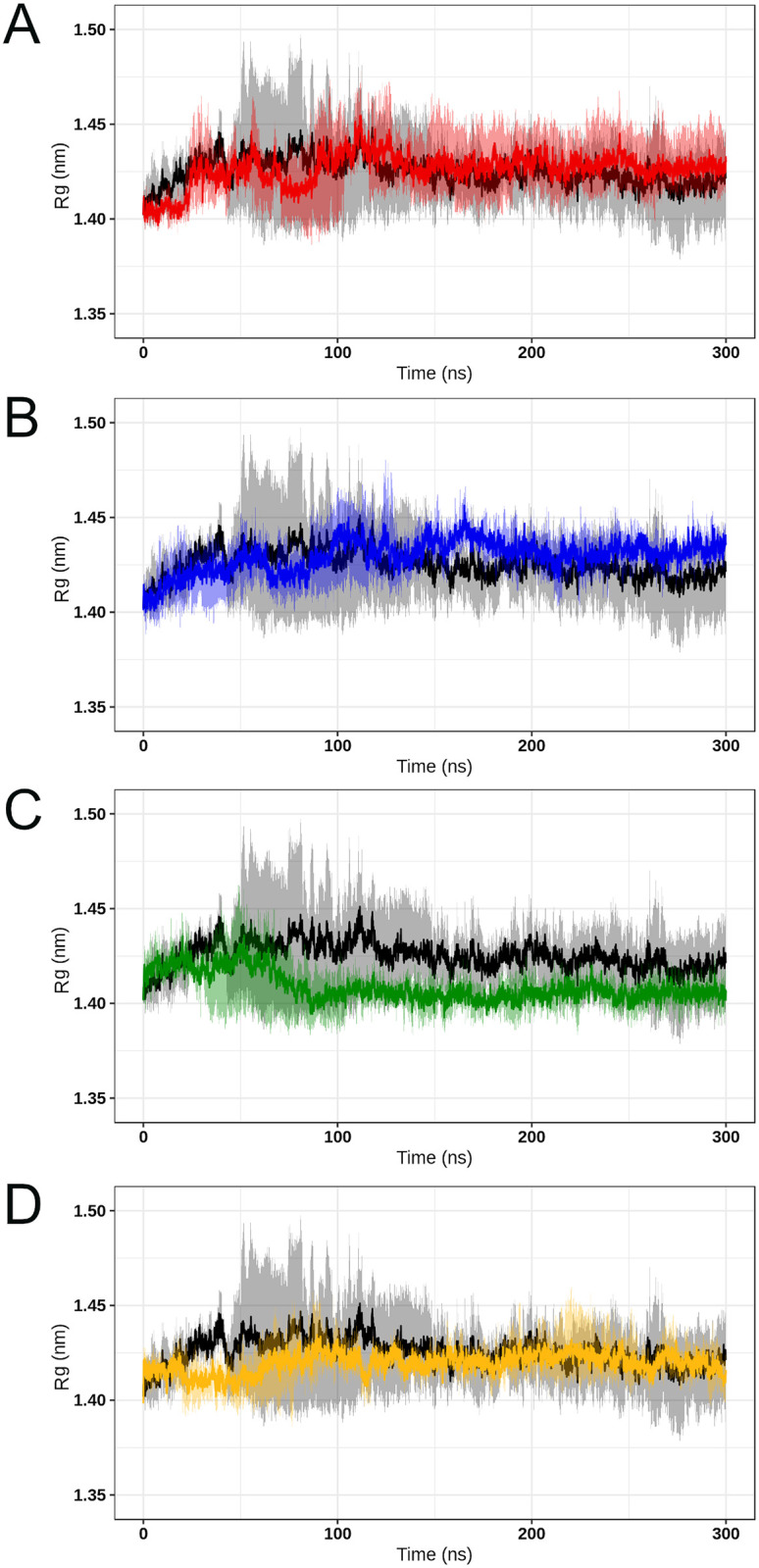
Rg analysis of wild-type SOD1 and its variants. The Rg values computed for the wild-type SOD1 and its variants at 300K are shown over time. The means (solid lines) and confidence intervals (smooth lines) are displayed for the triplicates. (A) The wild-type is represented in black, while variant A4V is represented in red. (B) The wild-type is represented in black, while variant D90A is represented in blue. (C) The wild-type is represented in black, while variant H46R is represented in green. (D) The wild-type is represented in black, while variant I113T is represented in yellow.

Rg is commonly used to measure the structural displacement of protein atoms from their common center of mass throughout the simulation, thus, providing comprehensive information on protein compactness over time [19]. The Rg values computed for the wild-type SOD1 and its variants pointed to an initial moment of structural instability, which is observed in the first half of all simulations (Fig 10). After this time, all the analyzed protein structures presented steady Rg values, which further suggests stable protein folding [54]. This analysis also indicated that the average Rg values for the wild-type SOD1 (1.422±0.016) are similar to that of variants A4V (1.428±0.015), D90A (1.433±0.01), H46R (1.405±0.008), and I113T (1.421±0.01). This result suggested no compactness alterations in the analyzed variants.

**Fig 11:**
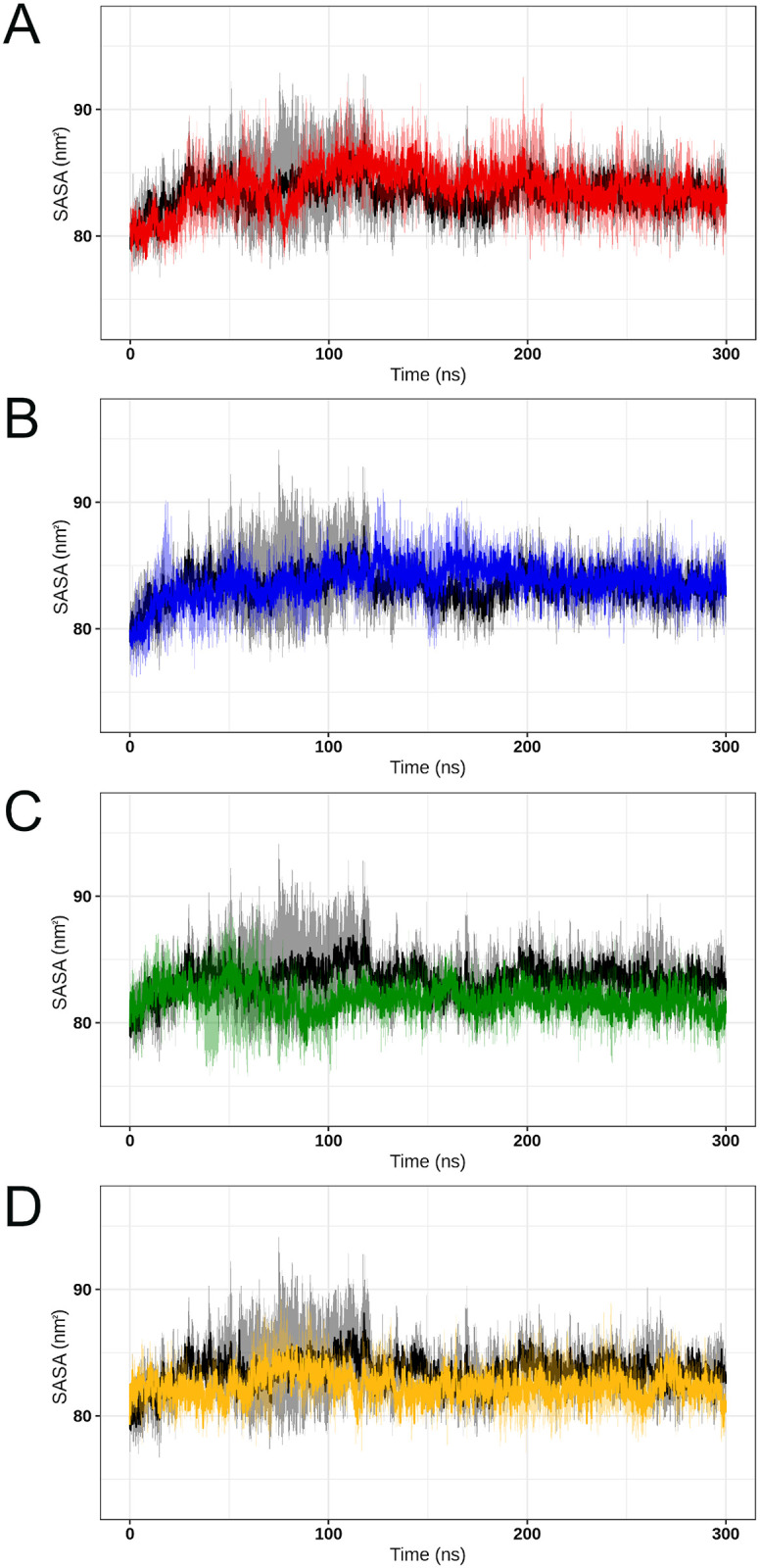
SASA analysis of wild-type SOD1 and its variants. The SASA values computed for the wild-type SOD1 and its variants at 300K are shown over time. The means (solid lines) and confidence intervals (smooth lines) are displayed for the triplicates. (A) The wild-type is represented in black, while variant A4V is represented in red. (B) The wild-type is represented in black, while variant D90A is represented in blue. (C) The wild-type is represented in black, while variant H46R is represented in green. (D) The wild-type is represented in black, while variant I113T is represented in yellow.

SASA is a measure of the exposed surface in protein structures that can be accessible to solvent molecules [55]. SASA analysis thus provides relevant information into the protein’s degree of exposure to its environment over time [56]. The SASA values computed throughout the simulations (Fig 11) pointed to an unstable behavior at the beginning of all simulations. After approximately 150ns, the SASA values assumed a steady behavior until the end of the MD trajectories. This analysis indicated that the average SASA values for the wild-type SOD1 (83.3±1.9) are similar to that of variants A4V (83.7±2.2), D90A (84.0±2.0), H46R (81.7±1.6), and I113T (82.2±1.8). This result suggested no SASA alterations in the analyzed variants.

**Fig 12:**
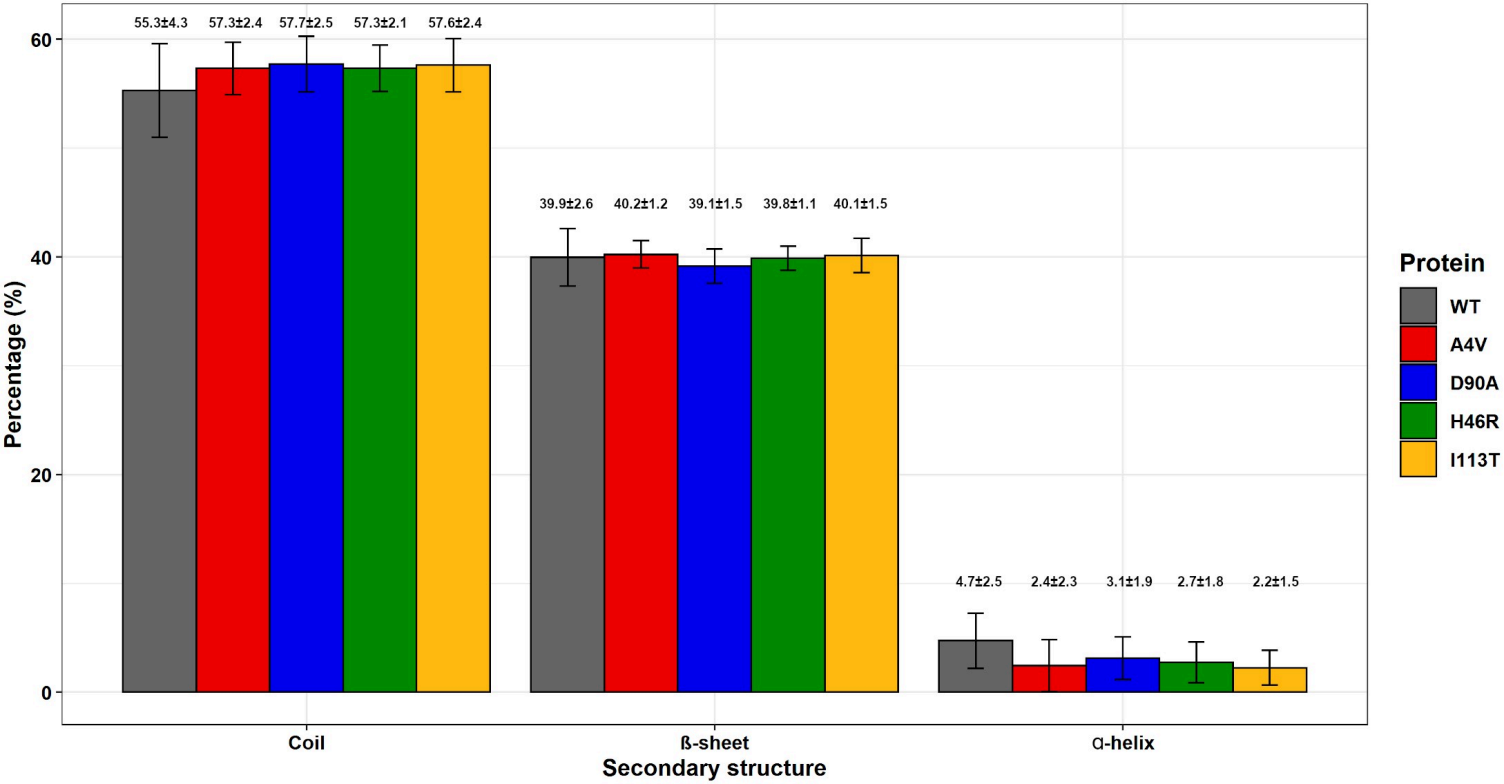
Secondary structure analysis of wild-type SOD1 and its variants. The average number (in percentages) of alpha-helices, beta-sheets, and coils formed throughout the simulations of wild-type SOD1 and its variants are shown as a bar plot. The error bar represents the standard deviation. The bar plot was designed using the ggplot2 library in R software. The average numbers and their respective standard deviations are also shown in the figure.

We also performed an SS analysis to further investigate the structural impact of SOD1 protein variants. The average number (in percentage) of coils, beta-sheets, and alpha-helices formed throughout the simulations was computed and displayed in barplot graphs. As shown in Fig 12, no alterations in the average number of SS were observed for the analyzed variants when compared to the wild-type SOD1.

## Discussion

Here, we compiled 233 missense mutations in the SOD1 protein (S1 Table), including thirty mutations that have not yet been registered in the dbSNP database. Forty-seven percent of these variants were classified as deleterious by all the functional prediction algorithms used (Fig 2D), suggesting that they might be harmful to SOD1. Our findings also suggested that missense mutations in SOD1 are most likely to be harmful than neutral to protein function, given the elevated rates of deleterious predictions computed for the variants (Fig 2D). Furthermore, the SOD1 variants with the highest rates of deleterious predictions in the functional prediction analysis mainly affect its metal-binding and electrostatic loops (S1 Fig), which reinforces the functional importance of the amino-acids within these regions.

The functional prediction algorithms used (S2 Fig) presented high accuracy in detecting the known functional impact of the variants A4V, H46R, and I113T [15, 39]. However, these algorithms presented low accuracy in predicting the deleterious potential of variant D90A. Thus, as previously shown by our group, using a variety of algorithms is crucial for predicting the functional impact of missense mutations. Especially because these algorithms apply different strategies to make predictions, and no gold standard method is currently established for this purpose [16].

Our findings also suggested that stability reduction is a common feature in SOD1 protein variants, given the elevated rate of destabilizing predictions (forty-nine percent) in the Mutant3.0 and Fold-X analyses (S3 Table). *In vitro* studies analyzing the stability of over 100 SOD1 protein variants have shown that most of these mutations are destabilizing [57, 58].

The divergent results observed at the stability prediction of some SOD1 protein variants (S3 Table) may occur due to the different strategies applied by FoldX and I-Mutant to make predictions. FoldX applies an empirical force field trained in a database of engineered proteins to estimate free-energy changes (ΔΔG) upon mutations and evaluate the stability impact of missense mutations [59], while I-Mutant 2.0 uses Support Vector Machine and information from a database of experimentally determined structures for this purpose [60].

This analysis also showed that the variants A4V, D90A, and I113T were predicted to decrease protein stability by Fold-X and I-Mutant, which is in agreement with the literature consulted [61–65]. The variant H46R, in turn, was only predicted as destabilizing by Fold-X (S3 Table). The variant H46R is not destabilized relative to the wild-type SOD1 in the nascent state. After the post-translational modifications necessary for SOD1 activity [66], this variant becomes relatively destabilized [64, 67].

Four regions with high aggregation potential, comprised between the amino-acids 4–8, 100–106, 111–120, and 146–153, were predicted for SOD1 by Castillo and Ventura, 2009. Our findings showed that 14 variants (K3E, A4F, A4V, C6Y, C6W, C6F, D96V, R115G, R115C, H120L, C146R, G147C, G147R, G147A) were predicted to increase the SOD1 aggregation tendency, while 7 variants (A145D, G147D, V148G, I149T, I151T, I151S, and A152P) were predicted to decrease this feature (S3 Table). All these variants occur at known aggregation-prone regions of SOD1, except for variants D96V and H120L.

Although this association is not yet fully understood [68], stability reduction and protein aggregation are considered synergistic risk factors for ALS severity and short survival time [69, 70]. All the variants predicted to increase protein aggregation tendency, except for variants D96V and H120L, were also predicted to decrease protein stability by at least one functional prediction algorithm used (S2 Table and S3 Table). This result thereby suggested that these variants may be potentially harmful to SOD1 and, consequently, valuable targets for future investigation.

The SNPEffect analysis (S3 Table) also showed that five variants were predicted to increase SOD1 amyloid propensity (V14A, G16C, G16A, G16S, and A89Y), while 5 variants were predicted to decrease this feature (V14M, Q15R, I17V, I18M, and N19S). Four amyloid-prone segments were identified *in silico* at the SOD1 sequence by Ivanova *et al*., 2014: 14–21, 30–38, 101–107, and 147–153. All the mutations predicted here to affect amyloid propensity, except for variant A90Y, occur in one of these regions, i.e. between amino-acids 14 and 21. Amyloid-like aggregates were observed *in vitro* for SOD1 variants occurring at these amyloid-prone regions [41, 71].

SOD1 folding and Cu/Zn acquisition are catalyzed by the copper chaperone for SOD1 (CCS). In the absence of CCS interaction, SOD1 is highly likely to remain inactive and unfolded, being degraded or accumulate in toxic aggregates [72]. The predictive analysis showed that the variant P66R decreases SOD1 chaperone binding (S3 Table). This mutation was previously found in an fALS patient, but the characterization of its effects has not yet been performed [73]. The amino-acid substitution P66R occurs at the metal-binding loop of SOD1 [74], which could hinder binding, preventing CCS action [75–77].

The variant A4V was predicted to increase protein aggregation (S3 Table), which is a known feature of this variant [78]. The variants D90A, H46R, and I113T were not predicted to affect protein aggregation, amyloid propensity, nor chaperone binding (S3 Table). However, studies have shown that these variants presented an increased tendency to form aggregates and amyloid-like fibrils [43, 67, 70, 79–84].

Functionally important amino-acids on proteins are usually conserved throughout evolution due to the high selective pressure [19]. Most of the analyzed SOD1 variants occur in conserved positions (Fig 3B), which might be harmful to SOD1 as they affect possibly important amino-acids to protein, especially the variants also predicted as deleterious by all the functional prediction algorithms used (S1 Fig). Mutations affecting conserved amino-acids within SOD1 can cause ALS, which suggests that even minor alterations in this protein may severely affect its structure and function [85].

The variants affecting highly conserved amino-acids mostly occur at the metal-binding and electrostatic loops (S2 Fig). The highly conserved amino-acids within the SOD1 structure are mainly located on these functionally important loops [43, 86], particularly at positions directly involved in metal-binding, superoxide guidance, and active site pocket formation [42, 43, 75].

As predicted by ConSurf (S2 Fig), the amino-acid substitution A4V and H46R occur at known conserved regions of SOD1 [21, 35, 87]. Although our results indicated that the variant I113T occurs in a variable position, this amino-acid is evolutionarily conserved in mammals [88]. The variant D90A, in turn, was predicted to affect an average conserved position. Unlike most ALS-related mutations in SOD1, D90A does not occur in a conserved amino-acid [65].

High fluctuations of RMSD values (Fig 4) were observed at the beginning of all simulations, which pointed to an initial moment of structural instability. This behavior is further confirmed by the high fluctuations of Rg (Fig 10), and SASA (Fig 11) observed in the first half of simulations. This might be attributed to the initial kinetic shock of molecular systems that occur in MD simulation processes [34]. The RMSD analysis also indicated that the protein structures fluctuate around average stable conformations after approximately 150ns (Fig 4), making sense to analyze local structural parameters, such as RMSF and B-factor [20]. The Rg analysis further suggested stable protein folding for SOD1 wild-type and its variants in the second half of trajectories (Fig 10). No alterations in the protein compactness (Fig 10), accessible surface (Fig 11), and secondary structure formation (Fig 12) were observed for the analyzed variants.

The essential mobility alterations observed in the ED analysis (Figs 7 and 8) occur at regions similar to those found altered in the RMSF (Fig 5) and B-factor (Fig 6) analyses, which mainly affect the metal-binding and electrostatic loops of the variants. Flexibility alterations can lead to strong and non-intuitive consequences for protein binding properties, as structural flexibility is determinant for binding affinity and specificity [89]. Moreover, the biological function of a protein is usually determined by its essential motions, especially those involved in protein interactions and binding to substrates [19]. Thus, the alterations observed during the MD simulations for the analyzed variants may affect SOD1 binding properties and, possibly, functional interactions, particularly at the metal-binding and electrostatic loops.

The ALS-SOD1 pathogenesis was initially believed to arise from the oxidative damage caused by reduced SOD1 activity, nevertheless, studies have further revealed that SOD1-null mice do not develop motor neuron disorder. On the other hand, transgenic mice expressing ALS-SOD1 mutant proteins become paralyzed despite possessing normal/elevated levels of SOD1 activity [90]. These pieces of evidence then suggest that SOD1 mutations lead to motor neuron death through toxic-gain of function rather than loss of enzymatic activity [91, 92].

The precise nature of ALS-SOD1 toxicity has not yet been fully elucidated. However, a substantial body of literature suggested that it arises from SOD1 misfolding and aggregation, which are common properties of ALS-SOD1 mutations and central events in ALS-SOD1 pathophysiology [85, 90, 93, 94]. More than 180 SOD1 coding mutations have already been described in the literature as occurring all over the protein and causing the same disease phenotype, ALS. Apparently, through similar toxic mechanisms, even mutations in SOD1 such as G93A, which cause changes as simple as the addition of a methyl group, can lead to the development of ALS [94, 95].

Many studies have been carried out to understand the molecular basis of ALS-SOD1 toxicity. The study of Elam *et*.*al*. 2003 assessed the structural impact of the S134N and H46R variants in SOD1 using X-ray crystallography [90]. The study of Banci *et*.*al*. 2005 evaluated the effect of S134N using nuclear magnet resonance methods (RMN) [35]. The study of Molnar *et*.*al*. 2009 analyzed the impact of 13 ALS-SOD1 mutations (A4V, L38V, G41S, H46R, G72Y, D76S, G85R, D90A, G93A, D124V, D125H, and S134N) on protein structure and dynamics using hydrogen/deuterium exchange mass spectrometry [95]. Despite the different methods used by the authors, all of them compared the ALS-related mutants with the wild-type protein and observed structural or dynamics perturbations at the electrostatic loop of the analyzed variants [35, 90, 95]. Molnar *et*.*al*. 2009 also observed alterations at the metal-binding loop of variants A4V, G93A, S134N, G85R, D124V, and D125H. No results were generated for the electrostatic and metal-binding loops of H46R in Molnar’s hydrogen/deuterium exchange experiments [95]. Further studies comparing the reactivity of conformation-specific antibodies for wild-type SOD1 and a series of other variants confirmed that SOD1 mutations induce some degree of misfolding at the electrostatic and metal-binding loop [85]. Together with the wet-lab experiments previously described in this paragraph, the molecular dynamics results presented here for A4V, D90A, H46R, and I113T further suggest that dynamics and structural alterations at the metal-binding and electrostatic loops could be a common characteristic of ALS-SOD1 mutations [35, 90, 95].

The computational methods are important allies of the wet-lab experiments [96], as the *silico* analyses can complement and overcome some difficulties of *in vitro* and *in vivo* assays [97, 98]. The MD simulations performed in this study can contribute to a better understanding of the molecular basis of ALS-SOD1 since this method provides detailed information on protein conformation and fluctuations over time [20], which can complement the results of already performed wet-lab experiments and overcome some limitations such as the static images provided by X-ray crystallography and the limited size of peptic fragments that can be analyzed by hydrogen/deuterium exchange [95]. On the other hand, we did not analyze whether SOD1 mutations not related to ALS also cause dynamics and structural alterations at the electrostatic and metal-binding loops, which is a limitation of this study. The alterations observed here and in the wet-lab experiments previously mentioned [35, 85, 90, 95] for ALS-SOD1 mutations could be an effect of SOD1 mutations in general, not necessarily related to the development of ALS.

Although the biological consequences of alterations at the functional loops of ALS-SOD1 variants are not fully understood, a well-accepted hypothesis suggests that this common property could lead to ALS possibly through a similar toxic mechanism involving aberrant interactions with cellular constituents and protein aggregation [68, 85, 90, 93, 95]. The study of Elam *et*.*al*. 2003 indicated that well-ordered electrostatic and metal-binding loops of SOD1 are critical to prevent protein aggregation. This study suggested that the functional loops adopt non-native conformations upon H46R and S134N mutations, which deprotects a hydrophobic core of the β-barrel region of SOD1 that serves as a molecular interface for non-native protein interactions. Among the non-native interactions described by Elam *et*.*al*. 2003, extensive apolar and hydrogen-bond interactions were formed between the electrostatic loop of a mutant protein and the deprotected β-barrel region of a neighboring SOD1 [90]. Banci *et*.*al*. 2005 identified abnormal contacts at the electrostatic loop and increased aggregate formation for the variant S134N in solution, which the authors attributed to the increased mobility and flexibility also observed at this functional loop. Protein surface regions characterized by high mobility and flexibility have been described as an important factor for molecular recognition [93]. Aberrant interactions with dynein and mitochondrial proteins, such as BCL-2, were also reported in the literature as a common feature of ALS-SOD1 mutations. The study of Zhang *et*.*al*. 2007 showed that the variants A4V, G85R, and G93A interact with the dynein, while the wild-type SOD1 does not. SOD1 mutant forms with disordered loop regions preferentially interact with the dynein complex, favoring aggregation [99]. Thus, together with these previous studies, our MD simulation results can further support this hypothesis since the flexibility and essential dynamics alterations observed at the functional loops of A4V, D90A, H46R, and I113T may favor or disfavor interactions, possibly leading to abnormal association with cellular constituents [19, 89].

## Conclusion

Two hundred and thirty-three SOD1 variants were compiled from the databases and literature consulted, which underwent functional and stability predictions. The predictive analysis pointed to an elevated rate of deleterious and destabilizing predictions for these variants, indicating their harmful effects. Among the analyzed variants, those occurring at the electrostatic and metal-binding loops were noticeably more damaging to protein structure and function, which makes them valuable targets for future investigation. The ConSurf analysis indicated that mutations in SOD1 mainly affect conserved and possibly functionally important amino-acids. The MD analyses of variants A4V, D90A, H46R, and I113T pointed to flexibility and essential dynamics alterations at the electrostatic and metal-binding loops that could lead to aberrant interactions triggering toxic protein aggregation. These alterations may have harmful implications for SOD1 and explain their association with ALS. Understanding the effects of SOD1 mutations on protein structure and function facilitates the design of further experiments and provides relevant information on the molecular mechanism of pathology, which may contribute to improvements in existing treatments for ALS.

## Supporting information

S1 Table SOD1 variants compiled from the literature (PubMed) and databases (ClinVar, UNIPROT, ALSod, dbSNP, and OMIM). (DOCX)

S2 Table Functional prediction for each SOD1 protein variant. (DOCX)

S3 Table Stability and SNPEffect4.0 predictions for each SOD1 protein variants. (DOCX)

S1 Fig Functional prediction of SOD1 protein variants. The SOD1 variants compiled from the literature and databases (233) were analyzed using ten different functional prediction algorithms. The bar plot indicates the number of neutral (gray) and deleterious (red) predictions for each SOD1 variant. The mutations predicted as deleterious by all the functional predictions algorithms used are marked by an asterisk. (A) Functional prediction from the variant A2V to L39V. (B) Functional prediction from the variant T40I to V88M. (C) Functional prediction from the variant A90V to V120L. (D) Functional prediction from the variant H121Q to A153P.
(TIF)

S2 Fig Evolutionary conservation analysis of SOD1 protein variants. The bar plot shows the ConSurf score for each amino-acid of SOD1 protein affected by mutations. The bar plot was colored according to the ConSurf coloring-scheme, which varies from cyan and variable to maroon and conserved. The mutations affecting highly conserved amino-acids (i.e. ConSurf score = 9) are marked by an asterisk. (A) ConSurf prediction from the variant A2V to L39V. ConSurf did not predict the conservation score of the A2V variant due to the lack of evolutionary information. (B) ConSurf prediction from the variant T40I to A90Y. (C) ConSurf prediction from the variant A90V to V120L. (D) ConSurf prediction from the variant H121Q to A153P.
(TIF)
