# Supplementary material for: Comprehensive in silico analysis and molecular dynamics of the superoxide dismutase 1 (SOD1) variants related to amyotrophic lateral sclerosis
Source: PLoS One. 2021 Feb 25;16(2):e0247841. doi: 10.1371/journal.pone.0247841 (PMC7906464; doi:10.1371/journal.pone.0247841)
Supplement: S1 Table — (DOCX) [file pone.0247841.s001.docx]

S1 Table. SOD1 variants compiled from the literature (PubMed) and databases (ClinVar, UNIPROT, ALSod, dbSNP, and OMIM).

| **Number** | **Amino acid position** | **Variant** | **dbSNP ID or article DOI*** |
| --- | --- | --- | --- |
| 1 | 2 | A1V/p.A2V | rs1297567794 |
| 2 | 4 | K3E/p.K4E | rs1568807297 |
| 3 | 5 | A4F/p.A5F | 10.1016/j.jns.2011.03.041 |
| 4 | 5 | A4D/p.A5D | https://doi.org/10.1002/ncn3.8 |
| 5 | 5 | A4S/p.A5S | rs121912444 |
| 6 | 5 | A4P/p.A5P | 10.1002/humu.22157** |
| 7 | 5 | A4T/p.A5T | rs121912444 |
| 8 | 5 | A4V/p.A5V | rs121912442 |
| 9 | 6 | V5L/p.V6L | rs1568807314 |
| 10 | 7 | C6Y/p.C7Y | rs121912448 |
| 11 | 7 | C6W/p.C7W | 10.1016/j.neurobiolaging.2013.08.024 |
| 12 | 7 | C6F/p.C7F | rs121912448 |
| 13 | 7 | C6G/p.C7G | rs1312702973 |
| 14 | 7 | C6S/p.C7S | rs1312702973 |
| 15 | 8 | V7M/p.V8M | rs1380854315 |
| 16 | 8 | V7E/p.V8E | rs1568807330 |
| 17 | 9 | L8Q/p.L9Q | rs1568807342 |
| 18 | 9 | L8V/p.L9V | rs1568807333 |
| 19 | 10 | K9Q/p.K10Q | rs1236713490 |
| 20 | 11 | G10A/p.G11A | rs1555836167 |
| 21 | 11 | G10V/p.G11V | rs1555836167 |
| 22 | 11 | G10R/p.G11R | rs1568807350 |
| 23 | 12 | D11Y/p.D12Y | rs762628133 |
| 24 | 12 | D11A/p.D12A | rs1568807374 |
| 25 | 13 | G12A/p.G13A | rs1379845624 |
| 26 | 13 | G12R/p.G13R | rs121912456 |
| 27 | 15 | V14A/p.V15A | rs1202989817 |
| 28 | 15 | V14G/p.V15G | rs1202989817 |
| 29 | 15 | V14M/p.V15M | rs1568807400 |
| 30 | 16 | Q15R/p.Q16R | rs200016533 |
| 31 | 17 | G16C/p.G17C | rs121912453 |
| 32 | 17 | G16A/p.G17A | rs1200906022 |
| 33 | 17 | G16S/p.G17S | rs121912453 |
| 34 | 18 | I17V/p.I18V | rs1460554436 |
| 35 | 19 | I18M/p.I19M | rs1182088847 |
| 36 | 20 | N19S/p.N20S | rs768029813 |
| 37 | 21 | F20L/p.F21L | rs1555836170 |
| 38 | 21 | F20V/p.F21V | 10.1186/s40035-018-0142-8 |
| 39 | 21 | F20C/p.F21C | rs1555836169 |
| 40 | 22 | E21G/p.E22G | rs1568807435 |
| 41 | 22 | E21Q/p.E22Q | 10.1016/j.neurol.2017.05.008 |
| 42 | 22 | E21K/p.E22K | rs121912450 |
| 43 | 23 | Q22L/p.Q23L | rs1169198442 |
| 44 | 23 | Q22H/p.Q23H | rs1424217272 |
| 45 | 23 | Q22R/p.Q23R | rs1169198442 |
| 46 | 26 | S25T/p.S26T | rs747214897 |
| 47 | 26 | S25N/p.S26N | rs747214897 |
| 48 | 29 | P28R/p.P29R | rs11556621 |
| 49 | 30 | V29A/p.V30A | rs1568809118 |
| 50 | 32 | V31G/p.V32G | rs1428716759 |
| 51 | 32 | V31A/p.V32A | rs1428716759 |
| 52 | 33 | W32G/p.W33G | 10.1186/s40035-018-0142-8 |
| 53 | 35 | S34I/p.S35I | rs777560607 |
| 54 | 36 | I35F/p.I36F | rs1057524474 |
| 55 | 38 | G37V/p.G38V | rs1555836517 |
| 56 | 38 | G37R/p.G38R | rs121912431 |
| 57 | 39 | L38R/p.L39R | rs1555836520 |
| 58 | 39 | L38Q/p.L39Q | rs1555836520 |
| 59 | 39 | L38V/p.L39V | rs121912432 |
| 60 | 40 | T39I/p.T40I | rs1804450 |
| 61 | 41 | E40G/p.E41G | rs1568809149 |
| 62 | 42 | G41D/p.G42D | rs121912434 |
| 63 | 42 | G41S/p.G42S | rs121912433 |
| 64 | 44 | H43R/p.H44R | rs121912435 |
| 65 | 46 | F45S/p.F46S | rs121912457 |
| 66 | 46 | F45C/p.F46C | rs121912457 |
| 67 | 47 | H46Y/p.H47Y | rs748897491 |
| 68 | 47 | H46D/p.H47D | rs748897491 |
| 69 | 47 | H46R/p.H47R | rs121912443 |
| 70 | 48 | V47A/p.V48A | rs1568809169 |
| 71 | 48 | V47F/p.V48F | rs1555836523 |
| 72 | 49 | H48Q/p.H49Q | rs1568809175 |
| 73 | 49 | H48R/p.H49R | rs1568809172 |
| 74 | 50 | E49V/p.E50V | rs770404622 |
| 75 | 50 | E49K/p.E50K | rs1568809178 |
| 76 | 51 | F50C/p.F51C | rs759149157 |
| 77 | 55 | T54R/p.T55R | rs986277034 |
| 78 | 58 | C57R/p.C58R | rs1568810255 |
| 79 | 60 | S59N/p.S60N | rs1413388444 |
| 80 | 60 | S59I/p.S60I | rs1413388444 |
| 81 | 61 | A60E/p.A61E | rs1378635853 |
| 82 | 62 | G61R/p.G62R | rs1568810268 |
| 83 | 65 | F64L/p.F65L | rs1030039318 |
| 84 | 66 | N65S/p.N66S | rs1568810275 |
| 85 | 67 | P66R/p.P67R | rs1568810284 |
| 86 | 67 | P66A/p.P67A | rs1356474292 |
| 87 | 67 | P66S/p.P67S | rs1356474292 |
| 88 | 68 | L67P/p.L68P | rs1568810289 |
| 89 | 68 | L67R/p.L68R | rs1568810289 |
| 90 | 69 | S68F/p.S69F | rs778327622 |
| 91 | 69 | S68Y/p.S69Y | rs778327622 |
| 92 | 70 | R69G/p.R70G | rs1457291290 |
| 93 | 72 | H71Y/p.H72Y | 10.1534/genetics.116.190850 |
| 94 | 73 | G72D/p.G73D | 10.1038/srep44606 |
| 95 | 73 | G72C/p.G73C | rs121912455 |
| 96 | 73 | G72S/p.G73S | rs121912455 |
| 97 | 74 | G73E/p.G74E | 10.1016/j.neurobiolaging.2018.01.013 |
| 98 | 74 | G73R/p.G74R | rs1555836720 |
| 99 | 75 | P74S/p.P75S | 10.1080/21678421.2019.1582668 |
| 100 | 77 | D76V/p.D77V | rs1568810316 |
| 101 | 77 | D76Y/p.D77Y | dx.doi.org/10.1038/srep32478 |
| 102 | 80 | R79S/p.R80S | 10.1186/s40035-018-0142-8 |
| 103 | 81 | H80A/p.H81A | 10.1002/ana.10369 |
| 104 | 81 | H80R/p.H81R | rs121912458 |
| 105 | 84 | D83N/p.D84N | rs1555836789 |
| 106 | 84 | D83G/p.D84G | rs1568810615 |
| 107 | 85 | L84F/p.L85F | rs1315541036 |
| 108 | 85 | L84V/p.L85V | rs121912452 |
| 109 | 86 | G85R/p.G86R | rs121912436 |
| 110 | 86 | G85C/p.G86C | 10.1186/s40035-018-0142-8 |
| 111 | 86 | G85S/p.G86S | rs121912436 |
| 112 | 87 | N86S/p.N87S | rs11556620 |
| 113 | 87 | N86K/p.N87K | rs1555836793 |
| 114 | 87 | N86I/p.N87I | rs11556620 |
| 115 | 87 | N86D/p.N87D | rs1555836792 |
| 116 | 88 | V87M/p.V88M | rs1568810641 |
| 117 | 88 | V87A/p.V88A | rs1339283341 |
| 118 | 90 | A89T/p.A90T | rs1568810660 |
| 119 | 90 | A89Y/p.A90Y | 10.1186/s40035-018-0142-8 |
| 120 | 90 | A89V/p.A90V | rs1280042397 |
| 121 | 91 | D90E/p.D91E | rs1256439749 |
| 122 | 91 | D90N/p.D91N | rs1343616996 |
| 123 | 91 | D90A/p.D91A | rs80265967 |
| 124 | 91 | D90V/p.D91V | rs80265967 |
| 125 | 92 | K91E/p.K92E | rs1345907062 |
| 126 | 93 | D92G/p.D93G | rs774994509 |
| 127 | 94 | G93A/p.G94A | rs121912438 |
| 128 | 94 | G93C/p.G94C | rs121912437 |
| 129 | 94 | G93D/p.G94D | rs121912438 |
| 130 | 94 | G93R/p.G94R | rs121912437 |
| 131 | 94 | G93S/p.G94S | rs121912437 |
| 132 | 94 | G93V/p.G94V | rs121912438 |
| 133 | 95 | V94G/p.V95G | 10.1186/s40035-018-0142-8 |
| 134 | 95 | V94A/p.V95A | rs202198235 |
| 135 | 96 | A95V/p.A96V | rs1568810690 |
| 136 | 96 | A95T/p.A96T | rs1568810686 |
| 137 | 96 | A95G/p.A96G | rs1568810690 |
| 138 | 97 | D96E/p.D97E | rs111229903 |
| 139 | 97 | D96V/p.D97V | rs1555836803 |
| 140 | 97 | D96N/p.D97N | rs121912459 |
| 141 | 98 | V97L/p.V98L | rs1555836806 |
| 142 | 98 | V97M/p.V98M | rs1555836806 |
| 143 | 100 | I99V/p.I100V | rs760740095 |
| 144 | 101 | E100G/p.E101G | rs121912439 |
| 145 | 101 | E100K/p.E101K | rs76731700 |
| 146 | 102 | D101G/p.D102G | rs1568810721 |
| 147 | 102 | D101H/p.D102H | rs1568810715 |
| 148 | 102 | D101Y/p.D102Y | rs1568810715 |
| 149 | 102 | D101E/p.D102E | 10.1016/j.neurobiolaging.2018.06.022 |
| 150 | 102 | D101N/p.D102N | rs1568810715 |
| 151 | 103 | S102A/p.S103A | rs936838632 |
| 152 | 105 | I104T/p.I105T | rs957386804 |
| 153 | 105 | I104F/p.I105F | rs121912445 |
| 154 | 106 | S105L/p.S106L | rs1378590183 |
| 155 | 107 | L106F/p.L107F | rs121912440 |
| 156 | 107 | L106V/p.L107V | rs121912440 |
| 157 | 107 | L106P/p.L107P | 10.1212/01.WNL.0000158679.47281.03 |
| 158 | 109 | G108E/p.G109E | rs1359299834 |
| 159 | 109 | G108R/p.G109R | rs1568810758 |
| 160 | 109 | G108V/p.G109V | rs1359299834 |
| 161 | 110 | D109N/p.D110N | rs567432143 |
| 162 | 110 | D109E/p.D110E | rs761014216 |
| 163 | 110 | D109Y/p.D110Y | rs567432143 |
| 164 | 112 | C111Y/p.C112Y | 10.1038/srep32478 |
| 165 | 113 | I112M/p.I113M | rs1299542356 |
| 166 | 113 | I112T/p.I113T | rs74315452 |
| 167 | 114 | I113M/p.I114M | rs750335577 |
| 168 | 114 | I113F/p.I114F | rs1568810780 |
| 169 | 114 | I113T/p.I114T | rs121912441 |
| 170 | 115 | G114A/p.G115A | rs1568810789 |
| 171 | 116 | R115G/p.R116G | rs1301635320 |
| 172 | 116 | R115H/p.R116H | rs1240334553 |
| 173 | 116 | R115C/p.R116C | rs1301635320 |
| 174 | 117 | T116R/p.T117R | rs1568810800 |
| 175 | 118 | L117V/p.L118V | rs199474723 |
| 176 | 119 | V118M/p.V119M | 10.1371/journal.pone.0160520 |
| 177 | 119 | V118L/p.V119L | rs1235629842 |
| 178 | 120 | V119F/p.V120F | 10.1038/srep44606 |
| 179 | 120 | V119L/p.V120L | rs1457889952 |
| 180 | 121 | H120Q/p.H121Q | 10.14336/AD.2019.0215 |
| 181 | 121 | H120R/p.H121R | rs1410925719 |
| 182 | 121 | H120L/p.H121L | rs1410925719 |
| 183 | 122 | E121G/p.E122G | rs1555836922 |
| 184 | 125 | D124A/p.D125A | rs1568811366 |
| 185 | 125 | D124G/p.D125G | rs1568811366 |
| 186 | 125 | D124V/p.D125V | rs1568811366 |
| 187 | 126 | D125H/p.D126H | rs1568811372 |
| 188 | 126 | D125A/p.D126A | rs1164911383 |
| 189 | 127 | L126S/p.L127S | rs121912454 |
| 190 | 128 | G127R/p.G128R | rs1568811389 |
| 191 | 130 | G129S/p.G130S | rs1464048449 |
| 192 | 131 | G130E/p.G131E | rs1169621300 |
| 193 | 132 | N131S/p.N132S | rs1447747586 |
| 194 | 133 | E132K/p.E133K | 10.1002/ana.24950 |
| 195 | 134 | E133G/p.E134G | 10.3109/21678421.2015.1107100 |
| 196 | 134 | E133V/p.E134V | rs1568811426 |
| 197 | 135 | S134T/p.S135T | rs121912451 |
| 198 | 135 | S134G/p.S135G | rs1555836932 |
| 199 | 135 | S134N/p.S135N | rs121912451 |
| 200 | 136 | T135I/p.T136I | rs781031581 |
| 201 | 138 | T137A/p.T138A | rs1568811445 |
| 202 | 138 | T137R/p.T138R | rs1568811454 |
| 203 | 139 | G138E/p.G139E | rs1568811464 |
| 204 | 140 | N139H/p.N140H | rs1568811471 |
| 205 | 140 | N139D/p.N140D | rs1568811471 |
| 206 | 140 | N139K/p.N140K | rs1804449 |
| 207 | 141 | A140T/p.A141T | rs1217353001 |
| 208 | 141 | A140G/p.A141G | rs1555836937 |
| 209 | 142 | G141A/p.G142A | rs1568811489 |
| 210 | 142 | G141R/p.G142R | 10.1136/jnnp-2015-311582 |
| 211 | 142 | G141E/p.G142E | rs1568811489 |
| 212 | 143 | S142N/p.S143N | rs1200970313 |
| 213 | 144 | R143C/p.R144C | rs746397967 |
| 214 | 144 | R143G/p.R144G | rs746397967 |
| 215 | 145 | L144F/p.L145F | rs1482760341 |
| 216 | 145 | L144S/p.L145S | rs121912446 |
| 217 | 146 | A145G/p.A146G | rs1131690781 |
| 218 | 146 | A145D/p.A146D | rs1131690781 |
| 219 | 146 | A145T/p.A146T | rs121912447 |
| 220 | 147 | C146R/p.C147R | rs1568811515 |
| 221 | 148 | G147D/p.G148D | rs1555836950 |
| 222 | 148 | G147S/p.G148S | 10.1038/sdata.2019.6 |
| 223 | 148 | G147C/p.G148C | 10.1136/jnnp-2013-307552 |
| 224 | 148 | G147R/p.G148R | rs1568811520 |
| 225 | 148 | G147A/p.G148A | 10.1111/ncn3.12153 |
| 226 | 149 | V148G/p.V149G | rs1476760624 |
| 227 | 149 | V148I/p.V149I | rs567511139 |
| 228 | 150 | I149V/p.I150V | rs1169917994 |
| 229 | 150 | I149T/p.I150T | rs1424014997 |
| 230 | 152 | I151T/p.I152T | rs121912449 |
| 231 | 152 | I151S/p.I152S | 10.1080/14660820310011700 |
| 232 | 153 | A152T/p.A153T | rs747094021 |
| 233 | 153 | A152P/p.A153P | 10.1016/j.neurobiolaging.2018.05.009 |

*Article DOI is shown when the mutation is not stored in the dbSNP database.

**The mutation A5P is only stored in the ALSod database.
