# Supplementary material for: Comprehensive in silico analysis and molecular dynamics of the superoxide dismutase 1 (SOD1) variants related to amyotrophic lateral sclerosis
Source: PLoS One. 2021 Feb 25;16(2):e0247841. doi: 10.1371/journal.pone.0247841 (PMC7906464; doi:10.1371/journal.pone.0247841)
Supplement: S3 Table — (DOCX) [file pone.0247841.s003.docx]

S3 Table. Stability and SNPEffect4.0 predictions for each SOD1 protein variants.

|  | **Stability Prediction** | | **SNPEffect4.0** | | |
| --- | --- | --- | --- | --- | --- |
| **Variant** | **I-Mutant3.0** | **Fold-X** | **TANGO** | **WALTZ** | **LIMBO** |
| A1V/p.A2V | decrease | decrease | does not affect | does not affect | does not affect |
| K3E/p.K4E | does not affect | decrease | increase | does not affect | does not affect |
| A4F/p.A5F | decrease | decrease | increase | does not affect | does not affect |
| A4D/p.A5D | decrease | decrease | does not affect | does not affect | does not affect |
| A4S/p.A5S | decrease | decrease | does not affect | does not affect | does not affect |
| A4P/p.A5P | does not affect | decrease | does not affect | does not affect | does not affect |
| A4T/p.A5T | decrease | decrease | does not affect | does not affect | does not affect |
| A4V/p.A5V | decrease | decrease | increase | does not affect | does not affect |
| V5L/p.V6L | increase | increase | does not affect | does not affect | does not affect |
| C6Y/p.C7Y | decrease | decrease | increase | does not affect | does not affect |
| C6W/p.C7W | does not affect | decrease | increase | does not affect | does not affect |
| C6F/p.C7F | decrease | decrease | increase | does not affect | does not affect |
| C6G/p.C7G | decrease | decrease | does not affect | does not affect | does not affect |
| C6S/p.C7S | decrease | decrease | does not affect | does not affect | does not affect |
| V7M/p.V8M | decrease | does not affect | does not affect | does not affect | does not affect |
| V7E/p.V8E | decrease | decrease | does not affect | does not affect | does not affect |
| L8Q/p.L9Q | decrease | decrease | does not affect | does not affect | does not affect |
| L8V/p.L9V | decrease | decrease | does not affect | does not affect | does not affect |
| K9Q/p.K10Q | does not affect | does not affect | does not affect | does not affect | does not affect |
| G10A/p.G11A | decrease | decrease | does not affect | does not affect | does not affect |
| G10V/p.G11V | decrease | decrease | does not affect | does not affect | does not affect |
| G10R/p.G11R | does not affect | decrease | does not affect | does not affect | does not affect |
| D11Y/p.D12Y | decrease | does not affect | does not affect | does not affect | does not affect |
| D11A/p.D12A | decrease | does not affect | does not affect | does not affect | does not affect |
| G12A/p.G13A | decrease | decrease | does not affect | does not affect | does not affect |
| G12R/p.G13R | does not affect | does not affect | does not affect | does not affect | does not affect |
| V14A/p.V15A | decrease | decrease | does not affect | increase | does not affect |
| V14G/p.V15G | decrease | decrease | does not affect | does not affect | does not affect |
| V14M/p.V15M | decrease | decrease | does not affect | decrease | does not affect |
| Q15R/p.Q16R | does not affect | does not affect | does not affect | decrease | does not affect |
| G16C/p.G17C | decrease | decrease | does not affect | increase | does not affect |
| G16A/p.G17A | decrease | decrease | does not affect | increase | does not affect |
| G16S/p.G17S | decrease | decrease | does not affect | increase | does not affect |
| I17V/p.I18V | decrease | decrease | does not affect | decrease | does not affect |
| I18M/p.I19M | decrease | decrease | does not affect | decrease | does not affect |
| N19S/p.N20S | does not affect | decrease | does not affect | decrease | does not affect |
| F20L/p.F21L | decrease | decrease | does not affect | does not affect | does not affect |
| F20V/p.F21V | decrease | decrease | does not affect | does not affect | does not affect |
| F20C/p.F21C | decrease | decrease | does not affect | does not affect | does not affect |
| E21G/p.E22G | decrease | decrease | does not affect | does not affect | does not affect |
| E21Q/p.E22Q | decrease | does not affect | does not affect | does not affect | does not affect |
| E21K/p.E22K | decrease | does not affect | does not affect | does not affect | does not affect |
| Q22L/p.Q23L | does not affect | decrease | does not affect | does not affect | does not affect |
| Q22H/p.Q23H | decrease | decrease | does not affect | does not affect | does not affect |
| Q22R/p.Q23R | does not affect | decrease | does not affect | does not affect | does not affect |
| S25T/p.S26T | does not affect | does not affect | does not affect | does not affect | does not affect |
| S25N/p.S26N | does not affect | does not affect | does not affect | does not affect | does not affect |
| P28R/p.P29R | decrease | decrease | does not affect | does not affect | does not affect |
| V29A/p.V30A | decrease | decrease | does not affect | does not affect | does not affect |
| V31G/p.V32G | decrease | decrease | does not affect | does not affect | does not affect |
| V31A/p.V32A | decrease | decrease | does not affect | does not affect | does not affect |
| W32G/p.W33G | decrease | decrease | does not affect | does not affect | does not affect |
| S34I/p.S35I | decrease | does not affect | does not affect | does not affect | does not affect |
| I35F/p.I36F | does not affect | decrease | does not affect | does not affect | does not affect |
| G37V/p.G38V | does not affect | decrease | does not affect | does not affect | does not affect |
| G37R/p.G38R | does not affect | decrease | does not affect | does not affect | does not affect |
| L38R/p.L39R | does not affect | decrease | does not affect | does not affect | does not affect |
| L38Q/p.L39Q | decrease | decrease | does not affect | does not affect | does not affect |
| L38V/p.L39V | does not affect | decrease | does not affect | does not affect | does not affect |
| T39I/p.T40I | decrease | does not affect | does not affect | does not affect | does not affect |
| E40G/p.E41G | decrease | does not affect | does not affect | does not affect | does not affect |
| G41D/p.G42D | does not affect | decrease | does not affect | does not affect | does not affect |
| G41S/p.G42S | does not affect | decrease | does not affect | does not affect | does not affect |
| H43R/p.H44R | does not affect | decrease | does not affect | does not affect | does not affect |
| F45S/p.F46S | decrease | decrease | does not affect | does not affect | does not affect |
| F45C/p.F46C | does not affect | decrease | does not affect | does not affect | does not affect |
| H46Y/p.H47Y | does not affect | decrease | does not affect | does not affect | does not affect |
| H46D/p.H47D | does not affect | decrease | does not affect | does not affect | does not affect |
| H46R/p.H47R | does not affect | decrease | does not affect | does not affect | does not affect |
| V47A/p.V48A | decrease | decrease | does not affect | does not affect | does not affect |
| V47F/p.V48F | decrease | decrease | does not affect | does not affect | does not affect |
| H48Q/p.H49Q | does not affect | decrease | does not affect | does not affect | does not affect |
| H48R/p.H49R | does not affect | decrease | does not affect | does not affect | does not affect |
| E49V/p.E50V | does not affect | increase | does not affect | does not affect | does not affect |
| E49K/p.E50K | does not affect | does not affect | does not affect | does not affect | does not affect |
| F50C/p.F51C | does not affect | decrease | does not affect | does not affect | does not affect |
| T54R/p.T55R | does not affect | does not affect | does not affect | does not affect | does not affect |
| C57R/p.C58R | does not affect | decrease | does not affect | does not affect | does not affect |
| S59N/p.S60N | does not affect | decrease | does not affect | does not affect | does not affect |
| S59I/p.S60I | does not affect | decrease | does not affect | does not affect | does not affect |
| A60E/p.A61E | does not affect | decrease | does not affect | does not affect | does not affect |
| G61R/p.G62R | does not affect | decrease | does not affect | does not affect | does not affect |
| F64L/p.F65L | decrease | does not affect | does not affect | does not affect | does not affect |
| N65S/p.N66S | does not affect | does not affect | does not affect | does not affect | does not affect |
| P66R/p.P67R | decrease | decrease | does not affect | does not affect | decrease |
| P66A/p.P67A | decrease | decrease | does not affect | does not affect | does not affect |
| P66S/p.P67S | decrease | decrease | does not affect | does not affect | does not affect |
| L67P/p.L68P | decrease | decrease | does not affect | does not affect | does not affect |
| L67R/p.L68R | decrease | does not affect | does not affect | does not affect | does not affect |
| S68F/p.S69F | does not affect | does not affect | does not affect | does not affect | does not affect |
| S68Y/p.S69Y | does not affect | does not affect | does not affect | does not affect | does not affect |
| R69G/p.R70G | decrease | decrease | does not affect | does not affect | does not affect |
| H71Y/p.H72Y | does not affect | decrease | does not affect | does not affect | does not affect |
| G72D/p.G73D | decrease | decrease | does not affect | does not affect | does not affect |
| G72C/p.G73C | decrease | decrease | does not affect | does not affect | does not affect |
| G72S/p.G73S | decrease | decrease | does not affect | does not affect | does not affect |
| G73E/p.G74E | does not affect | decrease | does not affect | does not affect | does not affect |
| G73R/p.G74R | does not affect | decrease | does not affect | does not affect | does not affect |
| P74S/p.P75S | decrease | decrease | does not affect | does not affect | does not affect |
| D76V/p.D77V | decrease | does not affect | does not affect | does not affect | does not affect |
| D76Y/p.D77Y | increase | does not affect | does not affect | does not affect | does not affect |
| R79S/p.R80S | decrease | decrease | does not affect | does not affect | does not affect |
| H80A/p.H81A | decrease | increase | does not affect | does not affect | does not affect |
| H80R/p.H81R | does not affect | increase | does not affect | does not affect | does not affect |
| D83N/p.D84N | decrease | decrease | does not affect | does not affect | does not affect |
| D83G/p.D84G | decrease | decrease | does not affect | does not affect | does not affect |
| L84F/p.L85F | decrease | decrease | does not affect | does not affect | does not affect |
| L84V/p.L85V | decrease | decrease | does not affect | does not affect | does not affect |
| G85R/p.G86R | does not affect | decrease | does not affect | does not affect | does not affect |
| G85C/p.G86C | decrease | decrease | does not affect | does not affect | does not affect |
| G85S/p.G86S | decrease | decrease | does not affect | does not affect | does not affect |
| N86S/p.N87S | does not affect | decrease | does not affect | does not affect | does not affect |
| N86K/p.N87K | decrease | does not affect | does not affect | does not affect | does not affect |
| N86I/p.N87I | increase | does not affect | does not affect | does not affect | does not affect |
| N86D/p.N87D | decrease | decrease | does not affect | does not affect | does not affect |
| V87M/p.V88M | decrease | decrease | does not affect | does not affect | does not affect |
| V87A/p.V88A | decrease | decrease | does not affect | does not affect | does not affect |
| A89T/p.A90T | decrease | decrease | does not affect | does not affect | does not affect |
| A89Y/p.A90Y | decrease | decrease | does not affect | increase | does not affect |
| A89V/p.A90V | decrease | decrease | does not affect | does not affect | does not affect |
| D90E/p.D91E | does not affect | decrease | does not affect | does not affect | does not affect |
| D90N/p.D91N | decrease | increase | does not affect | does not affect | does not affect |
| D90A/p.D91A | decrease | decrease | does not affect | does not affect | does not affect |
| D90V/p.D91V | decrease | decrease | does not affect | does not affect | does not affect |
| K91E/p.K92E | does not affect | decrease | does not affect | does not affect | does not affect |
| D92G/p.D93G | decrease | does not affect | does not affect | does not affect | does not affect |
| G93A/p.G94A | decrease | decrease | does not affect | does not affect | does not affect |
| G93C/p.G94C | does not affect | decrease | does not affect | does not affect | does not affect |
| G93D/p.G94D | decrease | decrease | does not affect | does not affect | does not affect |
| G93R/p.G94R | does not affect | decrease | does not affect | does not affect | does not affect |
| G93S/p.G94S | decrease | decrease | does not affect | does not affect | does not affect |
| G93V/p.G94V | does not affect | decrease | does not affect | does not affect | does not affect |
| V94G/p.V95G | decrease | decrease | does not affect | does not affect | does not affect |
| V94A/p.V95A | decrease | decrease | does not affect | does not affect | does not affect |
| A95V/p.A96V | does not affect | decrease | does not affect | does not affect | does not affect |
| A95T/p.A96T | decrease | decrease | does not affect | does not affect | does not affect |
| A95G/p.A96G | decrease | decrease | does not affect | does not affect | does not affect |
| D96E/p.D97E | does not affect | does not affect | does not affect | does not affect | does not affect |
| D96V/p.D97V | does not affect | does not affect | increase | does not affect | does not affect |
| D96N/p.D97N | decrease | does not affect | does not affect | does not affect | does not affect |
| V97L/p.V98L | decrease | decrease | does not affect | does not affect | does not affect |
| V97M/p.V98M | decrease | decrease | does not affect | does not affect | does not affect |
| I99V/p.I100V | decrease | decrease | does not affect | does not affect | does not affect |
| E100G/p.E101G | decrease | decrease | does not affect | does not affect | does not affect |
| E100K/p.E101K | decrease | increase | does not affect | does not affect | does not affect |
| D101G/p.D102G | decrease | decrease | does not affect | does not affect | does not affect |
| D101H/p.D102H | decrease | decrease | does not affect | does not affect | does not affect |
| D101Y/p.D102Y | does not affect | decrease | does not affect | does not affect | does not affect |
| D101E/p.D102E | does not affect | decrease | does not affect | does not affect | does not affect |
| D101N/p.D102N | decrease | decrease | does not affect | does not affect | does not affect |
| S102A/p.S103A | does not affect | does not affect | does not affect | does not affect | does not affect |
| I104T/p.I105T | decrease | decrease | does not affect | does not affect | does not affect |
| I104F/p.I105F | decrease | decrease | does not affect | does not affect | does not affect |
| S105L/p.S106L | does not affect | decrease | does not affect | does not affect | does not affect |
| L106F/p.L107F | decrease | decrease | does not affect | does not affect | does not affect |
| L106V/p.L107V | decrease | decrease | does not affect | does not affect | does not affect |
| L106P/p.L107P | decrease | decrease | does not affect | does not affect | does not affect |
| G108E/p.G109E | does not affect | decrease | does not affect | does not affect | does not affect |
| G108R/p.G109R | does not affect | decrease | does not affect | does not affect | does not affect |
| G108V/p.G109V | does not affect | decrease | does not affect | does not affect | does not affect |
| D109N/p.D110N | decrease | does not affect | does not affect | does not affect | does not affect |
| D109E/p.D110E | does not affect | does not affect | does not affect | does not affect | does not affect |
| D109Y/p.D110Y | does not affect | decrease | does not affect | does not affect | does not affect |
| C111Y/p.C112Y | decrease | decrease | does not affect | does not affect | does not affect |
| I112M/p.I113M | decrease | decrease | does not affect | does not affect | does not affect |
| I112T/p.I113T | decrease | decrease | does not affect | does not affect | does not affect |
| I113M/p.I114M | decrease | does not affect | does not affect | does not affect | does not affect |
| I113F/p.I114F | decrease | decrease | does not affect | does not affect | does not affect |
| I113T/p.I114T | decrease | decrease | does not affect | does not affect | does not affect |
| G114A/p.G115A | does not affect | decrease | does not affect | does not affect | does not affect |
| R115G/p.R116G | decrease | decrease | increase | does not affect | does not affect |
| R115H/p.R116H | decrease | decrease | does not affect | does not affect | does not affect |
| R115C/p.R116C | decrease | decrease | increase | does not affect | does not affect |
| T116R/p.T117R | does not affect | decrease | does not affect | does not affect | does not affect |
| L117V/p.L118V | decrease | decrease | does not affect | does not affect | does not affect |
| V118M/p.V119M | decrease | decrease | does not affect | does not affect | does not affect |
| V118L/p.V119L | decrease | decrease | does not affect | does not affect | does not affect |
| V119F/p.V120F | decrease | decrease | does not affect | does not affect | does not affect |
| V119L/p.V120L | increase | decrease | does not affect | does not affect | does not affect |
| H120Q/p.H121Q | does not affect | decrease | does not affect | does not affect | does not affect |
| H120R/p.H121R | does not affect | increase | does not affect | does not affect | does not affect |
| H120L/p.H121L | increase | increase | increase | does not affect | does not affect |
| E121G/p.E122G | decrease | decrease | does not affect | does not affect | does not affect |
| D124A/p.D125A | does not affect | decrease | does not affect | does not affect | does not affect |
| D124G/p.D125G | decrease | decrease | does not affect | does not affect | does not affect |
| D124V/p.D125V | decrease | decrease | does not affect | does not affect | does not affect |
| D125H/p.D126H | decrease | decrease | does not affect | does not affect | does not affect |
| D125A/p.D126A | does not affect | decrease | does not affect | does not affect | does not affect |
| L126S/p.L127S | decrease | decrease | does not affect | does not affect | does not affect |
| G127R/p.G128R | does not affect | decrease | does not affect | does not affect | does not affect |
| G129S/p.G130S | decrease | decrease | does not affect | does not affect | does not affect |
| G130E/p.G131E | does not affect | decrease | does not affect | does not affect | does not affect |
| N131S/p.N132S | does not affect | decrease | does not affect | does not affect | does not affect |
| E132K/p.E133K | decrease | decrease | does not affect | does not affect | does not affect |
| E133G/p.E134G | decrease | decrease | does not affect | does not affect | does not affect |
| E133V/p.E134V | does not affect | decrease | does not affect | does not affect | does not affect |
| S134T/p.S135T | does not affect | decrease | does not affect | does not affect | does not affect |
| S134G/p.S135G | does not affect | decrease | does not affect | does not affect | does not affect |
| S134N/p.S135N | does not affect | decrease | does not affect | does not affect | does not affect |
| T135I/p.T136I | does not affect | increase | does not affect | does not affect | does not affect |
| T137A/p.T138A | decrease | decrease | does not affect | does not affect | does not affect |
| T137R/p.T138R | does not affect | decrease | does not affect | does not affect | does not affect |
| G138E/p.G139E | does not affect | decrease | does not affect | does not affect | does not affect |
| N139H/p.N140H | does not affect | decrease | does not affect | does not affect | does not affect |
| N139D/p.N140D | does not affect | decrease | does not affect | does not affect | does not affect |
| N139K/p.N140K | does not affect | decrease | does not affect | does not affect | does not affect |
| A140T/p.A141T | decrease | decrease | does not affect | does not affect | does not affect |
| A140G/p.A141G | decrease | decrease | does not affect | does not affect | does not affect |
| G141A/p.G142A | does not affect | decrease | does not affect | does not affect | does not affect |
| G141R/p.G142R | does not affect | decrease | does not affect | does not affect | does not affect |
| G141E/p.G142E | does not affect | decrease | does not affect | does not affect | does not affect |
| S142N/p.S143N | does not affect | decrease | does not affect | does not affect | does not affect |
| R143C/p.R144C | does not affect | does not affect | does not affect | does not affect | does not affect |
| R143G/p.R144G | decrease | decrease | does not affect | does not affect | does not affect |
| L144F/p.L145F | decrease | decrease | does not affect | does not affect | does not affect |
| L144S/p.L145S | decrease | decrease | does not affect | does not affect | does not affect |
| A145G/p.A146G | decrease | decrease | does not affect | does not affect | does not affect |
| A145D/p.A146D | does not affect | decrease | decrease | does not affect | does not affect |
| A145T/p.A146T | decrease | decrease | does not affect | does not affect | does not affect |
| C146R/p.C147R | does not affect | decrease | increase | does not affect | does not affect |
| G147D/p.G148D | does not affect | decrease | decrease | does not affect | does not affect |
| G147S/p.G148S | decrease | decrease | does not affect | does not affect | does not affect |
| G147C/p.G148C | does not affect | decrease | increase | does not affect | does not affect |
| G147R/p.G148R | does not affect | decrease | increase | does not affect | does not affect |
| G147A/p.G148A | does not affect | decrease | increase | does not affect | does not affect |
| V148G/p.V149G | decrease | does not affect | decrease | does not affect | does not affect |
| V148I/p.V149I | decrease | increase | does not affect | does not affect | does not affect |
| I149V/p.I150V | decrease | decrease | does not affect | does not affect | does not affect |
| I149T/p.I150T | decrease | decrease | decrease | does not affect | does not affect |
| I151T/p.I152T | decrease | does not affect | decrease | does not affect | does not affect |
| I151S/p.I152S | decrease | decrease | decrease | does not affect | does not affect |
| A152T/p.A153T | decrease | decrease | does not affect | does not affect | does not affect |
| A152P/p.A153P | does not affect | decrease | decrease | does not affect | does not affect |
