# Supplementary figures and images for: Comprehensive in silico analysis and molecular dynamics of the superoxide dismutase 1 (SOD1) variants related to amyotrophic lateral sclerosis
Source: PLoS One. 2021 Feb 25;16(2):e0247841. doi: 10.1371/journal.pone.0247841 (PMC7906464; doi:10.1371/journal.pone.0247841)

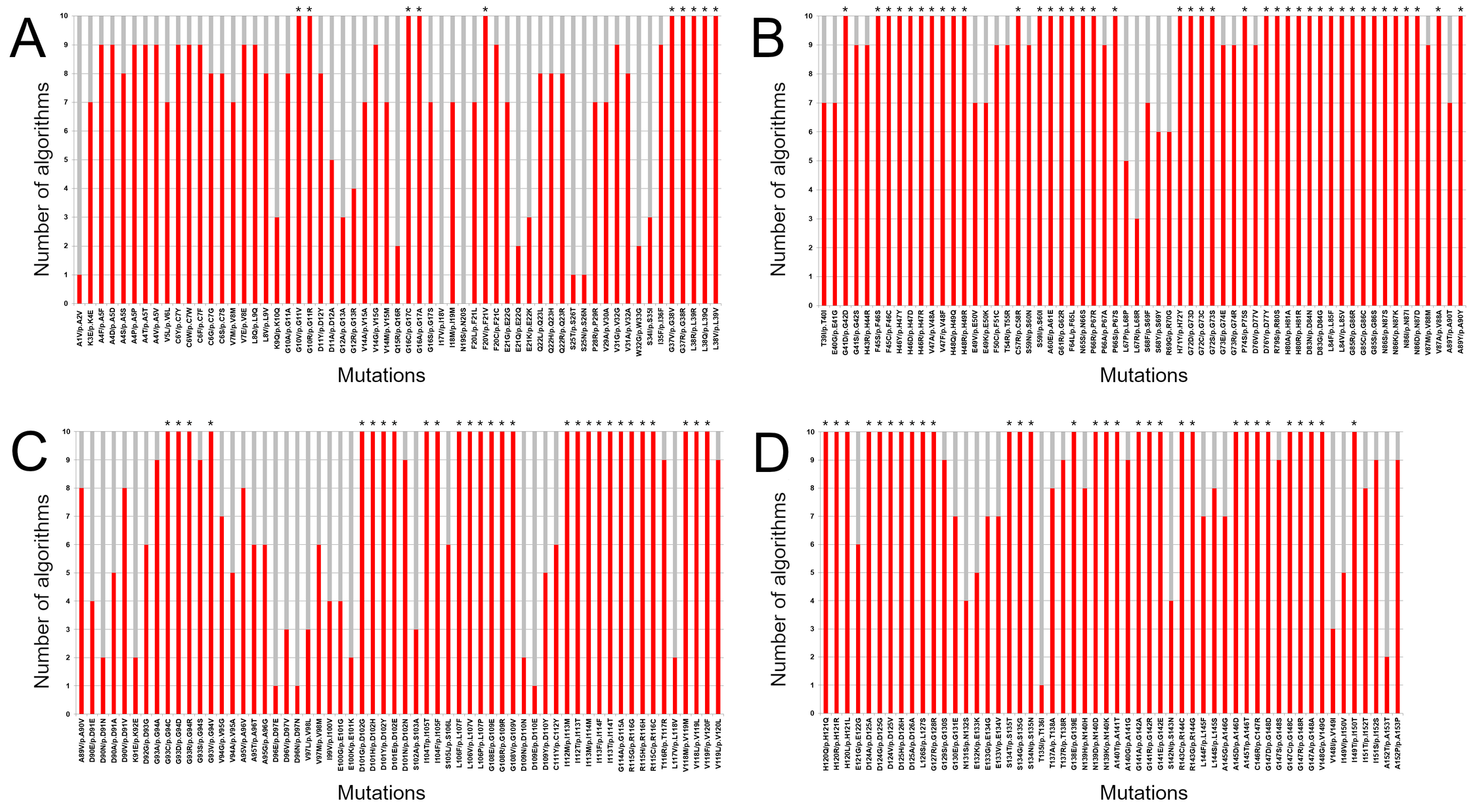

Supplement: S1 Fig — The SOD1 variants compiled from the literature and databases (233) were analyzed using ten different functional prediction algorithms. The bar plot indicates the number of neutral (gray) and deleterious (red) predictions for each SOD1 variant. The mutations predicted as deleterious by all the functional predictions algorithms used are marked by an asterisk. (A) Functional prediction from the variant A2V to L39V. (B) Functional prediction from the variant T40I to V88M. (C) Functional prediction from the variant A90V to V120L. (D) Functional prediction from the variant H121Q to A153P. (TIF) [file pone.0247841.s004.tif]

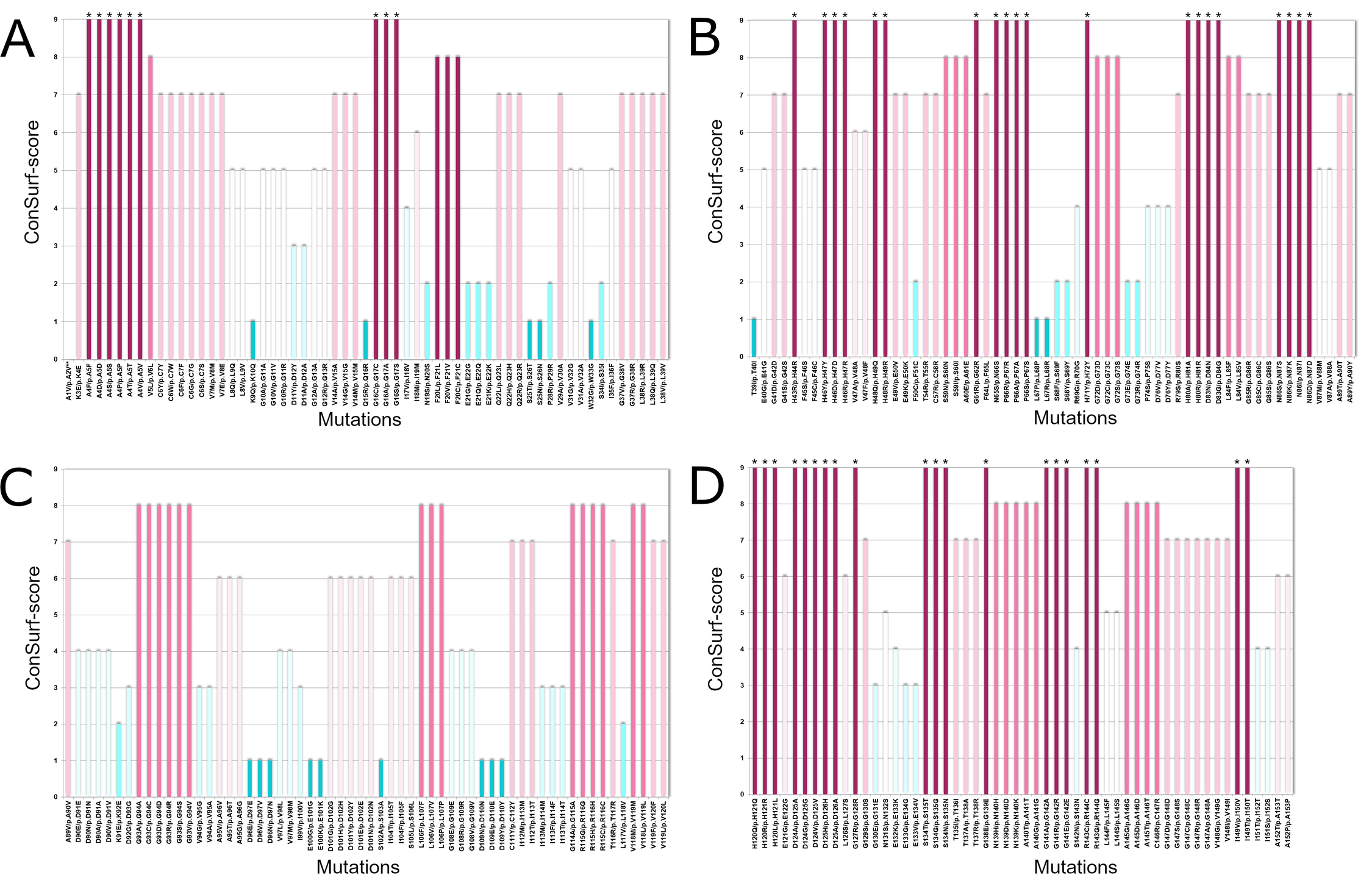

Supplement: S2 Fig — The bar plot shows the ConSurf score for each amino-acid of SOD1 protein affected by mutations. The bar plot was colored according to the ConSurf coloring-scheme, which varies from cyan and variable to maroon and conserved. The mutations affecting highly conserved amino-acids (i.e. ConSurf score = 9) are marked by an asterisk. (A) ConSurf prediction from the variant A2V to L39V. ConSurf did not predict the conservation score of the A2V variant due to the lack of evolutionary information. (B) ConSurf prediction from the variant T40I to A90Y. (C) ConSurf prediction from the variant A90V to V120L. (D) ConSurf prediction from the variant H121Q to A153P. (TIF) [file pone.0247841.s005.tif]
